# Medical importance and pharmacokinetics of gold nanoparticles in the human body

**DOI:** 10.1186/s12943-025-02418-3

**Published:** 2025-10-10

**Authors:** Priyanka Singh, Abhayraj S. Joshi, Hina Singh, Ivan Mijakovic

**Affiliations:** 1 The Novo Nordisk Foundation Center for Biosustainability, Technical University of Denmark, Lyngby, 2800 Denmark; 2 Manipal Centre for Biotherapeutics Research (MCBR), Manipal Academy of Higher Education (MAHE), Udupi, Karnataka 576104 India; 3 Division of Biomedical Sciences, School of Medicine, University of California, Riverside, CA 92521 USA; 4 Systems and Synthetic Biology Division, Department of Biology and Biological Engineering, Chalmers University of Technology, Gothenburg, Sweden

**Keywords:** Gold nanoparticles, Medical applications, Pharmacokinetics, Clinical trials, Biodistribution, Toxicity

## Abstract

Gold nanoparticles (AuNPs) have emerged as versatile nanocarriers with promising potential in therapeutic applications due to their tunable physicochemical properties, ease of functionalization, and excellent biocompatibility. Recognized as one of the most extensively studied nanomaterials, AuNPs have demonstrated utility in cancer diagnostics, photothermal therapy, targeted drug delivery, biosensing, vaccine development, antimicrobial treatments, gene delivery, imaging-guided therapy, and skin care formulations. While much research has focused on their design, formulations, and medical applications, understanding their fate within the human body is equally crucial. The long-term fate of AuNPs within the human body remains not well understood, largely due to the widely accepted notion that their inert nature impedes biodegradation. This review discusses the synthesis and biomedical applications of AuNPs, with a primary focus on recent advances in understanding their pharmacokinetic behavior in the human body guided by growing insights into their biological interactions, biodistribution, clearance mechanisms, and the key factors influencing there in vivo dynamics.

**Graphical abstract:**

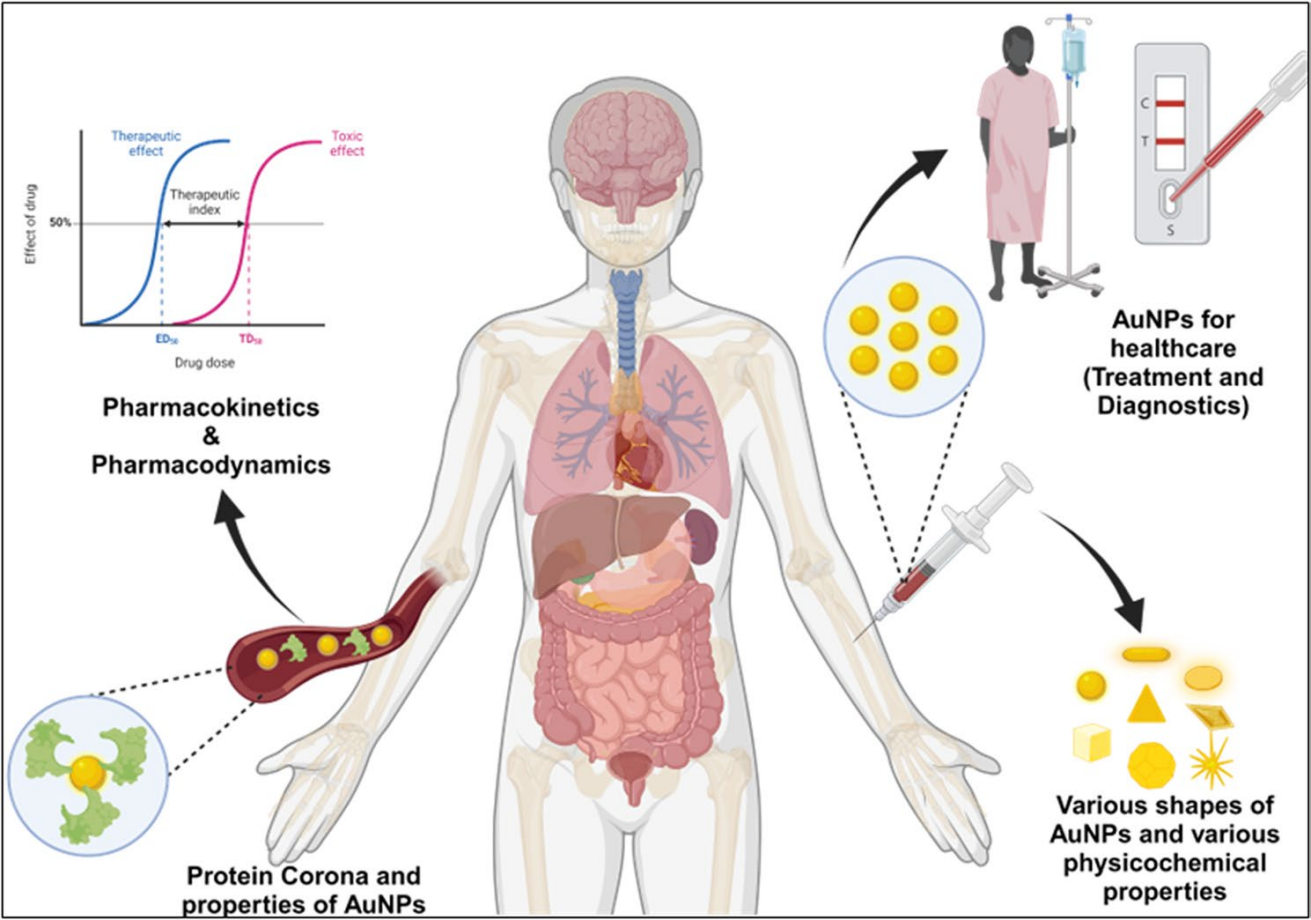

## Introduction

The medical field has been utilizing nanomaterials to tackle various chronic diseases, which were previously difficult to manage with traditional treatments alone, such as cancer, inflammation, rheumatoid arthritis, implants, neurodegenerative disorders, cardiovascular diseases, diabetes, and infectious diseases [1, 2]. Various nanomaterials were employed to address the shortcomings of these treatments, aiming to improve efficacy, reduce side effects, and enhance targeted delivery. Among these nanomaterials, gold nanoparticles (AuNPs) have emerged as key player due to their exceptional nanoscale properties [3, 4]. These properties comprise surface plasmon resonance (SPR), high surface-area-to-volume ratio, tunable size, shape, and the potential for surface functionalization [5, 6]. Such versatility enables AuNPs to find applications such as drug delivery, bioimaging, and diagnosis via biosensing across diverse disease conditions that include infections, cancer, diseases caused due to genetic mutations, metabolic disorders and various neurological disorders [7–10].

The distinctive SPR of AuNPs, which is significantly influenced by quantum size effects arising from electron confinement, allows precise manipulation of light and underpins their unique optical behavior. Additionally, SPR properties are highly sensitive to and can be modulated by particle size, surface charge, hydrophobicity, and shape, allowing the resonance peak to be tuned to specific wavelengths of light, an essential feature for their application in biosensing, imaging, and photothermal therapy (PTT) [11, 12]. In parallel, the high surface-area-to-volume ratio of AuNPs enhances their reactivity and molecular interactions, further supporting their widespread biomedical applications, particularly in drug delivery, biosensing and diagnostic fields [13]. Beyond these intrinsic properties, the ability to fine-tune the size, shape, and surface chemistry of AuNPs substantially expands their versatility in targeted medical applications [14]. AuNPs can be synthesized in a range of sizes, from ultrasmall particles (1–5 nm) to larger forms (20–100 nm), with this tunability proving especially critical in overcoming physiological barriers such as the blood–brain barrier (BBB) [15]. In addition to size, the diverse geometries of AuNPs, such as nanorods, nanospheres, triangles, and nanoclusters confer unique optical, mechanical, and biological properties that can be leveraged for selective targeting and enhanced transport across such barriers [16]. AuNPs-based nanocarriers have been engineered to deliver a wide array of payloads, including small molecules, proteins, and nucleic acids, enabling localized treatment, even within the brain [17]. Moreover, combining AuNPs with other nanomaterials such as liposomes, polymeric nanoparticles, or micelles facilitates the design of hybrid systems optimized for drug delivery, imaging, and therapeutic precision [18]. When integrated with strategies like focused ultrasound-mediated BBB disruption, these systems demonstrate improved brain penetration and spatiotemporal drug release, offering a promising avenue for the targeted treatment of neurological disorders. Furthermore, the exceptional environmental stability of AuNPs under varying pH, temperature, and ionic conditions ensures consistent performance and long-term reliability, reinforcing their suitability for a wide range of biomedical applications.

In addition to facilitating brain-targeted delivery, AuNPs-based systems have also shown promise in cancer therapy when engineered for controlled and stimuli-responsive drug release. While AuNPs are not inherently capable of releasing drugs in a controlled manner, their surfaces can be functionalized with smart polymers, oligonucleotides, or cleavable linkers that enable precise, on-demand drug activation in response to internal or external stimuli [19]. For example, near-infrared (NIR) light can trigger photothermal conversion, resulting in localized heating that promotes drug release and tumor ablation. Internally, the tumor microenvironment (TME) offers cues such as acidic pH, elevated redox potential, and enzymatic activity, which can be exploited through pH-sensitive or enzyme-cleavable linkers to achieve selective intratumoral release [20, 21]. This functionality is particularly advantageous in the treatment of solid tumors, where unique physiological characteristics such as leaky vasculature and impaired lymphatic drainage contribute to the enhanced permeability and retention (EPR) effect, enabling preferential nanoparticle accumulation. However, factors like elevated interstitial fluid pressure and heterogeneous perfusion can still hinder deep tissue penetration and lead to uneven drug distribution. In this context, stimuli-responsive AuNPs systems offer a strategic advantage by improving intratumoral targeting, enhancing therapeutic efficacy, and minimizing systemic toxicity. Moreover, functionalizing AuNPs surfaces with ligands that recognize tumor-specific receptors further increases the precision of both drug delivery and diagnostic imaging, advancing their potential for translation into clinical oncology [22]. In addition, functionalization of AuNPs surfaces with various biomolecules also represents a powerful strategy to enhance biocompatibility and circulation time within the body. More precisely, functionalization with polymers helps in improving biocompatibility and circulation time, whereas that with peptides, oligonucleotides, and antibodies help in targeting the tumors [23, 24].

Despite the numerous benefits and established applications of AuNPs in medical science, questions regarding the safety and toxicity of AuNPs are always raised [27]. One crucial aspect to consider is what happens after achieving drug delivery within the body. Do these nanostructures remain within the body, or are they eliminated through natural body’s excretion mechanisms? In addition, the concentration of AuNPs in various tissues and organs is essential for determining any potential accumulation or adverse effects of long-term exposure [28, 29]. Thus, understanding the in vivo fate of AuNPs post-delivery is essential for assessing their long-term safety and potential adverse effects.

**Fig. 1:**
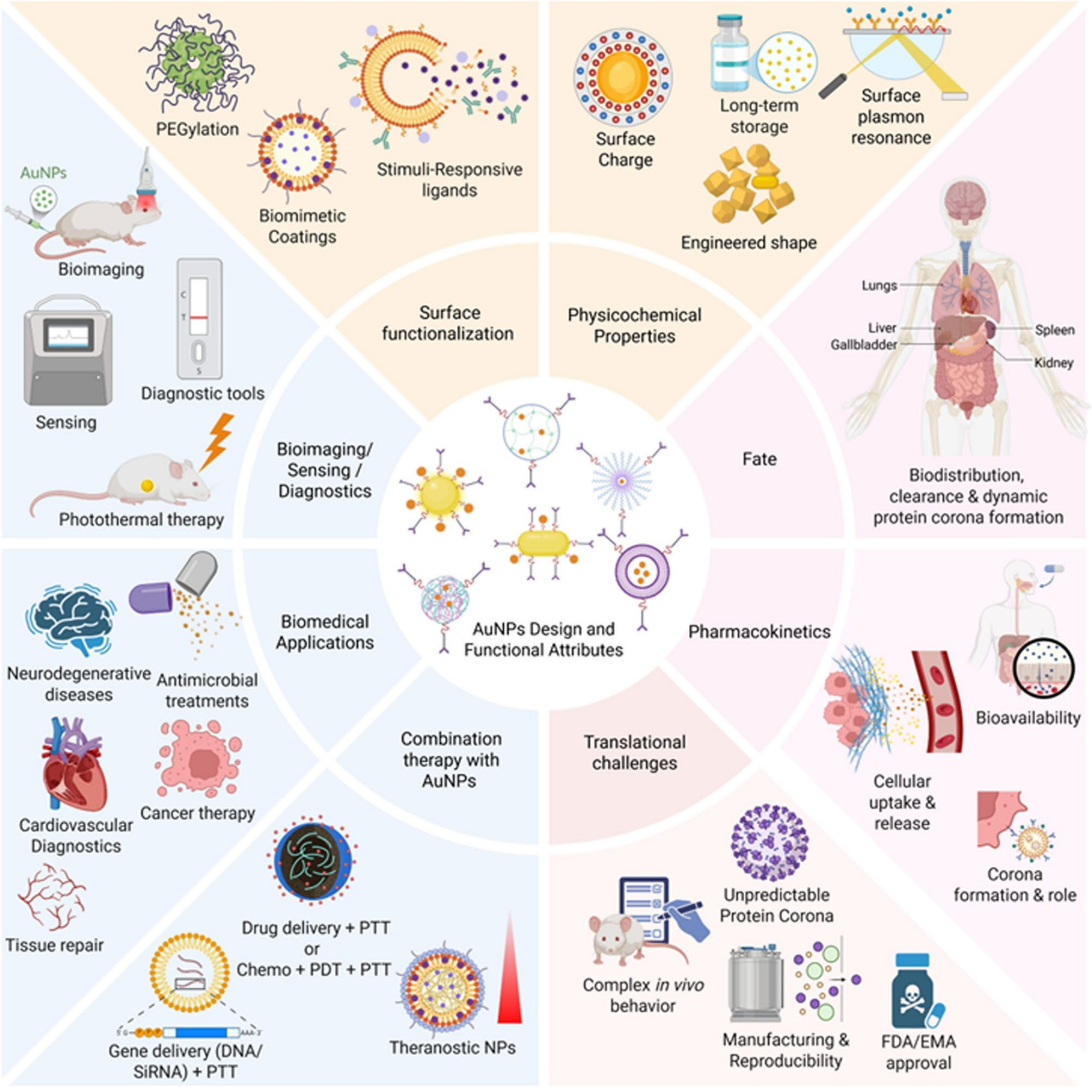
Overview of AuNPs in medical science. Illustration summarizes the critical properties, therapeutic applications, pharmacokinetics, and clinical relevance of AuNPs. These include roles in drug delivery, imaging, biosensing, and combination therapy. The figure also highlights the challenges in clinical translation, such as biodistribution, clearance, and protein corona formation. (Made by using biorender)

This comprehensive review thoroughly explores AuNPs, covering their synthesis, diverse medical applications and pharmacokinetics profile. Beginning with an overview of AuNPs fabrication and synthesis methodologies, the review lays the groundwork for understanding how nanoparticles can be tailored to address various medical needs. Moving beyond synthesis, the review elaborates on the details of drug delivery, imaging, and biosensing applications facilitated by AuNPs. However, despite promising reports from preclinical trials showcasing the therapeutic potential of AuNPs, their limited clinical success raises significant questions. By examining these translational barriers such as biological complexity, immune modulation, and clearance we aim to identify the key challenges obstructing broader clinical adoption. Figure 1 provides an integrated schematic that summarizes these interconnected aspects, illustrating the design considerations, biological interactions, therapeutic applications, and translational hurdles that define the current landscape of AuNPs in medicine.

## Methods of synthesis for AuNPs

**Fig. 2:**
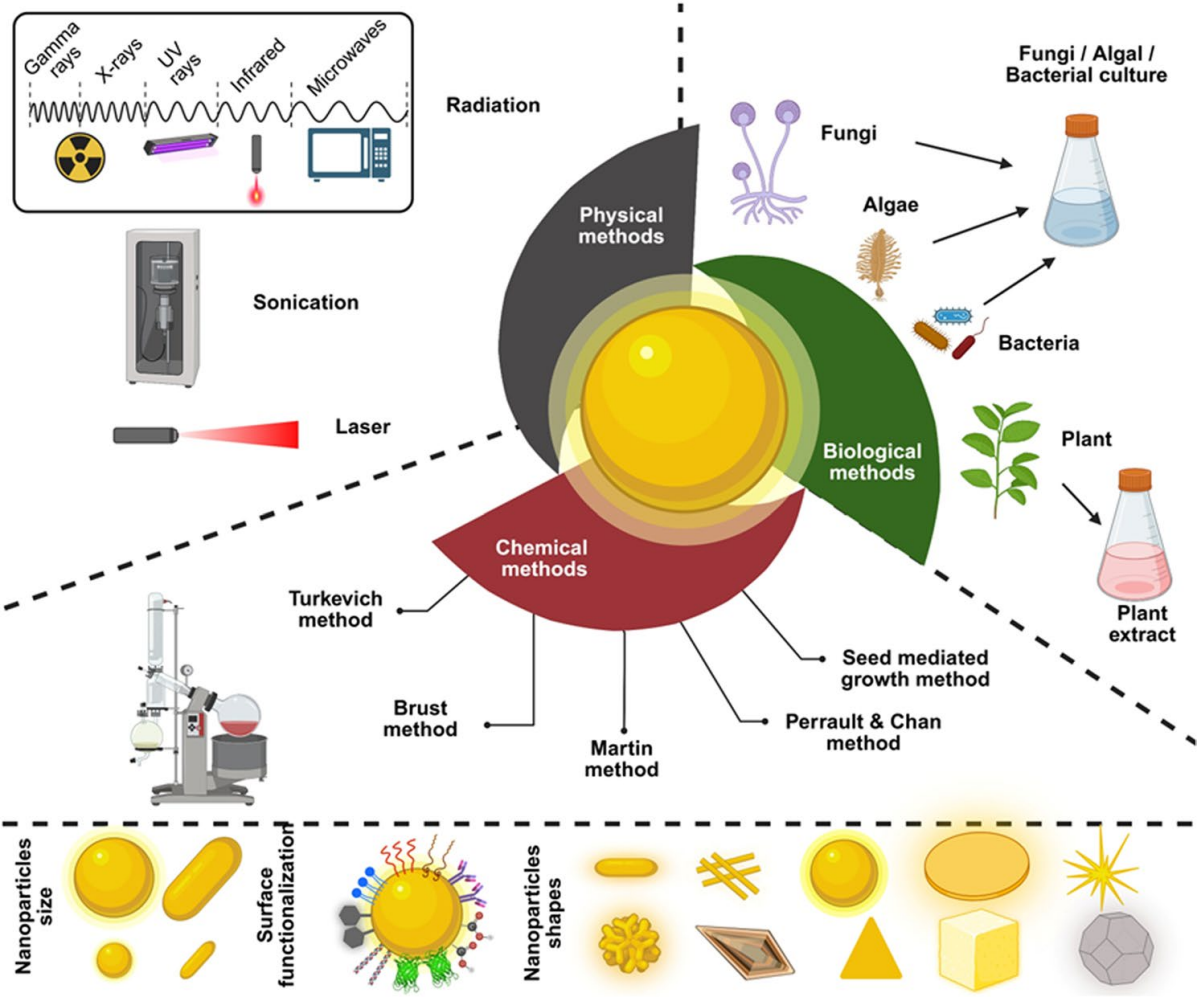
Overview of synthesis strategies used to produce AuNPs, organized into three main categories: physical, chemical, and biological methods. Techniques such as laser ablation, sonication, the Turkevich and Brust reduction processes, and green synthesis using plant extracts or microbial cultures are illustrated. Each method influences the resulting nanoparticle’s size, shape, and surface characteristics, which in turn affect their biological interactions and potential applications in medicine. (Created using biorender)

The preliminary stage of “synthesis of AuNPs” represents an important phase as it defines their properties and has direct influence on their final application. For AuNPs, the synthesis methods are broadly categorized into two classes: Top-down approaches and bottom-up approaches. In top-down approaches, the bulk gold material is converted to AuNPs with the help of sputtering technique, electric arc dispersion technique, lithography, and laser ablation method. However, these methods suffer with the drawback of non-uniformity, poor AuNPs stability, and high cost. However, bottom-up approaches overcome these drawbacks and generate reproducible, uniform, and stable AuNPs in a cost-effective way. The choice of bottom-up synthesis methodology significantly affects the properties of final gold nanoformulations in terms of their size, shape, stability, efficacy, physicochemical properties, etc. Hence, the choice of synthesis procedure has prime importance. For a given synthesis method, an array of variables such as the concentration of gold salt, stabilizers, catalysts such as acids or alkalis, various reducing agents, buffers, time of synthesis, and external power sources (if used), play an essential role in the developmental stage. Several methods and variants of these methods have been developed and used to prepare AuNPs. Broadly, these bottom-up synthesis methods can be categorized into three main classes: (i) Chemical methods, (ii) Physical methods, and (iii) Biological methods (Fig. 2) [30, 31].

### Chemical methods

The original method of AuNPs synthesis dates back to 1951 when Turkevich et al. described the efficient and reproducible synthesis of colloidal gold suspension and since then, the synthesis methodologies have evolved to a larger extent. These methods use a range of several polar and non-polar solvents and reducing agents to synthesize AuNPs of various sizes and shapes. Turkevich et al. showed that among several reducing agents such as citric acid, oxalic acid, acetylene, hydroxylamine, tannin solution, sodium citrate provided stable and spherical AuNPs at boiling temperature [32]. Over the time, several other acids such as ascorbic acid, squaric acid, croconic acid, and rhodizonic acid, have been successfully employed to prepare stable spherical AuNPs even at room temperature [33]. Afrapoli et al. [34] and Sivaraman et al. [35] used same reagents and performed reversed addition to show that such change in method yielded similar results. Panariello et al. recently modified the conventional Turkevich synthesis method by passivating gold precursor using 2 M NaOH solution in the first step and then mixed it with citrate for reduction and conversion into highly stable low size AuNPs. However, the downside of this method is the requirement of flow cells and additional reactors that increases the cost of the overall method [36].

Brust et al. used liquid-liquid biphasic system (water: toluene) in 1994 to synthesize ultrasmall AuNPs (1–5 nm) [37]. In the biphasic system, the gold salt from the aqueous layer moves to organic layers in the presence of a surfactant such as tetraoctylammonium bromide (TOAB), and the sodium borohydride (NaBH_4_) present in the solution help in reducing the gold salt to gold atoms leading to their growth into nanoparticles. The authors used alkanethiol moiety (in the organic layer) for the stabilization of AuNPs [37]. Recent advances in microfluidics have allowed this method to prepare AuNPs systematically, and reproducibly [38].

The two-step seed-mediated growth method is another common strategy to synthesize AuNPs of various shapes at either room temperature or elevated temperatures [39–45]. In the first nucleation step, the gold precursor is converted to gold clusters below 10 nm in size. Then, in the second growth step, reduced gold species (zero-valent gold atoms) are then allowed to deposit on the nuclei/seeds for the growth of the nanoparticles. In this method, at the nucleation step, hexadecyltrimethylammonium bromide (CTAB) or its derivatives act as shape directing agent that helps uniform dispersion and distribution of the gold seeds in the aqueous solution; whereas NaBH_4_ acts as a fast reducing agent that converts the gold species into ultrafine gold seeds. On the other hand, in the growth phase, several slow-reducing agents, such as citrate, ascorbate, polyampholytes, etc., are used [39–41]. Using this method, fairly good control has been achieved on the anisotropy of the AuNPs that have resulted in the synthesis of cubes, rods, pyramids, discs, triangles, and cage-shaped AuNPs [39]. However, the downside of this method is an increased probability of the formation of secondary, smaller particles due to unpredictable growth on the seeds as well as the adsorption of significant concentrations of CTAB that are toxic to human cells [18]. All aforementioned chemical methods utilize aqueous solvents to synthesize the AuNPs. Contrary to this, in 2010, Martin et al. showed the synthesis of charged AuNPs in the non-polar organic solvent by their biphasic separation [46]. Muhan Cao et al. used this strategy to prove long-term stability and superior dispersity of AuNPs in a range of polar and non-polar solvents. Authors also claimed that such wide dispersion ability may be useful for various applications of such AuNPs [47].

### Physical methods

These methods employ an additional external force to convert gold ions into nanoparticles. Such additional input components may be (A) Ultrasonic waves, (B) Radiation sources (X-ray radiation, Gamma radiation, UV radiation, Microwaves), or (C) Laser ablation. Several reports in the literature suggest that the energy provided by ultrasound waves creates transient regions of high pressure and high temperature with very fast and slow cooling rates within the liquid [48, 49]. Therefore, this method is usually used with chemical methods to produce low size AuNPs in a faster and easier way [50, 51]. However, it requires a special energy source that makes it an expensive method.

In the radiation assisted synthesis, the common radiation sources used are X-rays, gamma rays, microwaves, and UV rays. These radiation resources cause their effect in direct as well as indirect ways. These radiation sources can transfer energy to gold ions in the aqueous solution, leading to their reduction. On the other hand, these radiation sources also create free radicals such as hydroxyl radicals, hydrogen radicals, and superoxide radicals that can reduce gold ions. In both direct as well as indirect cases, the reduction of gold ions leads to the formation of ultrasmall AuNPs (below 10 nm) as well as small AuNPs (10–100 nm) [30]. For example, Zharikov et al. showed that X-rays originating from a 5-BKhV-6 W tube with a tungsten anode can be used for the synthesis of ultrasmall AuNPs (1–5 nm). Since the authors used solutions of two different polymers, these polymers stabilized the final AuNPs [52]. Miguel Toro-González et al. combined a novel millifluidic reactor platform with X-ray irradiator to synthesize AuNPs in the size range of 1–35 nm. The authors demonstrated the synthesis of the AuNPs within an hour at room temperature. They also stated that such synthesis platform can help in reducing the overall concentrations of reducing and stabilizing agents required to prepare stable ultrasmall AuNPs [53]. In a different report, Phan Ha Nu Diem et al. used gamma radiation for the synthesis of dextran-stabilized AuNPs. Authors attributed the nanoparticle formation process to the reduction of gold ions to their zero valent state owing to the energy provided by gamma rays upon irradiation [54]. The advantages of gamma irradiation method for synthesis of AuNPs are cleaner production, no need for excessive stabilizer or reductant, and capability of production of AuNPs at large scale.

Among all radiation-assisted synthesis methods, the UV irradiation-mediated AuNPs synthesis also known as photochemical synthesis, is the most commonly used method [55]. Several reports are available that show use of UV light in the synthesis of AuNPs [55–58]. Till date, among the three types of UV lights (UVA, UVB, and UVC) that differ in wavelength, UVC light has been used often [59, 60]. But UV light irradiation is not the only factor that governs the production of AuNPs. The pH of solvents being used, type of reducing agents present in the vicinity of gold ions, the temperature, and irradiation time also affect the final nanoparticle product significantly [56]. Along with UV light, a variety of molecules such as polymers, proteins, non-ionic surfactants, small molecules, and amino acids have been used to coat the nanoparticles for better stability and/or improved pharmacological properties [57, 59, 61, 62]. UV irradiation method can also be combined with conventional chemical methods [63] as well as the biological method [58, 64]. In general, UV irradiation methods are fast, reproducible, and versatile techniques that can be combined with chemical and biological methods to produce the AuNPs; however, poor control on shape of the nanoparticles as well as requirement of special dedicated UV light source makes this method less applicable.

Microwave-assisted synthesis represents the fourth type of radiation-based method. Similar to UV assisted methods, this method can also be combined with conventional chemical synthesis methods [65, 66] and the biological methods [67, 68]. The basic advantage of microwave assisted method is use of household and cost-effective microwave devices to heat the reaction mixture. Furthermore, the microwaves do not affect the nanoparticles that could lead to change in their properties [65]. In this method, the chemical component (such as citrate) or the biological component (biomolecules) perform dual function as reducing agent as well as stabilizing agents to convert the gold precursor into AuNPs in presence of thermal energy provided by the irradiated microwaves. Instant heating provided by microwaves helps in yielding uniform sized nanoparticles. Just like UV irradiation method, microwave assisted method has been combined with the state of the art continuous flow reactor for continuous and bulk production of ultrasmall AuNPs (1–4 nm) in reproducible manner [69]. Overall, among all radiation-based methods, microwave assisted methods pose as cheaper option but still need dedicated equipment of the synthesis.

Laser Ablation is another physical method, which, within short duration produce the AuNPs of varying sizes and shapes [70, 71]. In this method, the gold target immersed in the aqueous solution of stabilizer or surfactant is irradiated using a wavelength laser. Upon irradiation, the thermal energy provided by laser source leads to ejection of gold atoms or their clusters which cool down in surrounding aqueous solvent in the form of nanoparticles [70]. However, due to sudden ejection and cooling, the anisotropy is introduced that results in particles having varying sizes and shapes [71]. Various other factors that influence this anisotropy are laser source energy, ablation energy, volume of solvent, ablation time, number of pulse used in ablation or pulse frequency, use of stabilizer or surfactant [71–73]. Hence, owing to the poor control of particle size and particle size distribution, this method is not commonly used.

The microplasma is another source with which AuNPs of different shapes and sizes can be synthesized. It has been shown that microplasma is generated using a cathode and anode (gold metal foil) while keeping both electrodes in a solution of gold salt and stabilizer. In presence of discharge produced upon induction of microplasma, the high energy electrons as well as free radicals are generated that reduces gold ions into atomic gold which further in absence or presence of reductant and stabilizer (e.g. citrate) get converted to AuNPs [26]. For example, Cai et al. synthesized citrate stabilized ultrasmall AuNPs (< 5 nm) using microplasma with discharge voltage of 2.7 kV. In this report, authors stated that if nebulization is used near the interface of microplasma and aqueous phase, the process yields uniform and smaller particles instead of generation of large aggregates [74]. Based on same principle, Kim et al. successfully synthesized monodispersed AuNPs as well as gold-silver core shell nanoparticles without use of any reducing agent or stabilizer with the help of microplasma [75]. These reports also suggest that microplasma assisted synthesis yields ultrasmall AuNPs with highly stable and monodispersed samples. However, to generate microplasma, a setup of electrodes with provision of a neutral gas (e.g. Helium) is necessary. Such setup increased the overall cost of the method.

Even though the chemical and physical synthesis methods yield AuNPs in the desired range of size as well as shape, these methods are not feasible at all times. Furthermore, these methods suffer from serious drawbacks of the requirement of hazardous chemicals, specialized equipment, external energy source, trained personnel, specialized laboratory setup which outweigh their utility in the mass production of AuNPs. Due to these drawbacks, the processing of the AuNPs obtained from these methods becomes cumbersome, especially when those AuNPs have application in the biomedical field. Therefore, the search for alternate synthesis methodologies has resulted in establishing several biological methods. In these methods, one can use microorganisms (such as bacteria or fungi), (B) algae, and (C) plants for the synthesis of AuNPs with various sizes and shapes.

### Biological methods

Microorganisms such as bacteria and fungi are one of the best resources for synthesizing AuNPs. They offer cheap and ecofriendly options owing to no need for hazardous and/or toxic chemicals, no high energy demand, no specialized dedicated equipment, and easy maintenance [76]. Bacteria or fungi, when subjected to metal ion stress, try to detoxify it. One way to do so is to reduce the metal ion species present in the surrounding environment with the help of several enzymes and/or metabolites. The result of this reduction process is the formation of AuNPs. Both bacteria and fungi, can form AuNPs either intracellularly or extracellularly. In case of intracellular synthesis of AuNPs, the gold ions released from the gold precursor get internalized through the bacterial cell membrane owing to the electrostatic interactions and the cytoplasmic proteins/enzymes/polysaccharides reduce these ions to form the AuNPs. For the extracellular synthesis of AuNPs, the proteins or polysaccharides secreted by the bacterial/fungal cells are employed [77]. Logically, the extracellular synthesis is easier and cheaper than intracellular one because the purification steps for obtaining AuNPs trapped within cells is tedious process. However, intracellular synthesis of AuNPs has its own advantages in terms of natural provision as well as abundant availability of co-enzymes, signaling molecules, co-factors, energy factor molecules such as NADH in the cell. Mimicking such synthesis machinery in vitro will make the overall process overwhelmingly expensive [78]. Till date, several bacterial species have shown to produce AuNPs of spherical, triangular, hexagonal, cubic, oval, or irregular shape in the size range of 5–200 nm [79, 80]. Some bacteria capable of withstanding extreme environmental conditions such as *Deinococcus radiodurans*, *Geobacillus sp.strain ID17*, *Bacillus sp. isolate GL1.3*, *Geobacillus wiegelii strain GWE1* have also shown successful conversion of gold precursor to AuNPs. The main advantage of using such bacteria is that they allow change of several variables such as temperature, pH, radiation power to wider range for obtaining AuNPs having various different properties (size and shape). One cannot apply such wider range of variables to other normal bacteria [81–85].

Apart from bacteria, fungi are the other source from which AuNPs can be obtained upon feeding them with gold precursors [86–89]. Several fungi have been shown to synthesize AuNPs having size in the Wide range of 1–300 nm and a variety of shapes (e.g., triangles, decahedral, Hexagonal, rods, irregular, ellipsoidal, 2D nanowires, pentagonal, nanowalls, spiral plates, icosahedral, spherical etc.) [88–90]. In fungal cells, the AuNPs can be synthesized on the surface, i.e. cell wall, or within the cytoplasmic membrane, extracellularly, or intracellularly. Zsófia Molnár et al. have shown applicability of 29 strains of thermophilic fungi in the production of AuNPs of various sizes. Notably, while working on these many strains, the authors pointed out the main Limitation of the biological methods which is, the identification of main components that are responsible for formation and stabilization of the AuNPs. The authors also stated that the size distribution among the AuNPs synthesized from 29 fungi strains showed standard deviation of almost 30–70% [91]. The AuNPs with wide standard deviation in size distribution may not be ideal for biomedical applications as the physicochemical properties may change owing to a change in the size [92]. Yet, Zsófia Molnár et al. demonstrated that if right strain and right method parameters are chosen, fungi pose as an excellent alternative for the biological production of AuNPs [91]. It is true that the microbial strain and species determine the final properties of AuNPs; however, several other factors such as precursor concentration, pH of the media, the type of the media used for microbial/fungal growth, temperature, and time of incubation matter to a significant extent.

The algal biomass is also capable of converting gold precursors to defined shaped AuNPs intracellularly or extracellularly. Several reports suggest that algal biomass can be used in its dry powder form or ethanolic extract form in order to synthesize the AuNPs. Till date, several brown, red, and green algae from various families have shown successful synthesis of AuNPs [79, 80, 93, 94].

 In addition, plants represent a prime component of nature that has enormous impact of human health and they are another excellent source from which the AuNPs can be produced. In many cases, the parts of plants such as leaves, stem, bark, fibers (separated from stem), roots, flower or flower petals, fruits, peels, rhizomes etc. are used in production of AuNPs [76, 95]. The extracts prepared from these plant parts using hot water or water-alcohol mixtures contain several biological components like polysaccharides, monosaccharides (sugars), proteins, peptides, amino acids, secondary metabolites such as flavonoids, alkaloids, glycosides that can participate in reduction of gold precursors to form the AuNPs and also offer stability by capping their surface [96]. Several plants from various families have been used for the production of AuNPs [80, 97–110]. The comparison between AuNPs obtained from plant sources and chemically synthesized AuNPs yielded vital information about their size and stability [111]. It has been shown that owing to fast reduction and capping by aforementioned phytochemicals, the AuNPs obtained from plants show lower size and higher stability than chemically synthesized counterparts [98]. Moreover, these nanoparticles can be easily optimized and obtained with a desired morphology by tuning the process parameters such as temperature, pH, salt concentration, etc [112].

Each synthesis method, whether it be a chemical, physical, or biological, has unique advantages and challenges that make it suitable for applications (Table 1). The choice of method depends on the specific requirements of the intended application, with considerations for factors such as particle size, shape, stability, and biocompatibility. The biggest advantages of biological methods are their environmentally friendly nature and cheaper production cost for AuNPs. Unlike physical and chemical methods, these methods do not produce any hazardous by-products. Moreover, these methods do not require additional energy sources like physical methods. Biological methods do not need hazardous organic solvents. Due to the usage of natural resources, these methods are also called as “green” methods [111]. Owing to the “green” nature, these methods also aid the biosustainability and green industry initiative started by several regulatory authorities across the globe. While biological synthesis methods are often promoted as eco-friendly and less toxic due to their avoidance of harsh chemicals, it is important to recognize that these methods are not entirely free from limitations. Biological entities such as plant extracts, fungi, or bacterial metabolites can introduce organic residues or surface-bound impurities that may affect nanoparticle stability and purity. These biomolecules may adsorb onto the surface of AuNPs and potentially lead to batch variability or immunogenic responses if not adequately purified. Conversely, some physical methods, including laser ablation, evaporation-condensation, or microplasma synthesis, can offer high-purity nanoparticles without the use of toxic solvents or reducing agents. These methods, although often energy-intensive and costly, may serve as clean alternatives for applications demanding stringent purity levels. Thus, careful method selection and downstream purification are essential regardless of the synthesis approach.

**Table 1:** Comparison of different synthesis methods of AuNPs, highlighting their size control, stability, scalability, biomedical suitability, and environmental impact, providing insights into their optimal application for medical use

Method	Size Control	Stability	Scalability	Biomedical Suitability	Sustainability	Key Insights
Chemical (Turkevich, Brust, Seed-mediated)	Moderate–High (via precursor & reducing agent control)	High (PEGylation improves)	Scalable	Good (widely used in drug delivery, imaging)	Low (toxic solvents, surfactants)	Enables surface modification; reproducible
Physical (Laser ablation, Microwave, UV radiation)	High (controlled via pulse width, energy)	Moderate	Limited (high energy required)	Limited (few surface ligands, bio-inert)	Moderate	Produces clean, surfactant-free particles
Biological (Bacteria, Fungi, Plant extracts)	High (via enzymatic/biomolecule control)	High	Scalable	Excellent (natural capping agents, low toxicity)	High (green synthesis)	Mechanism varies; slower reactions (some microorganisms based); Fast (mostly plants based)

## Applications for AuNPs

**Fig. 3:**
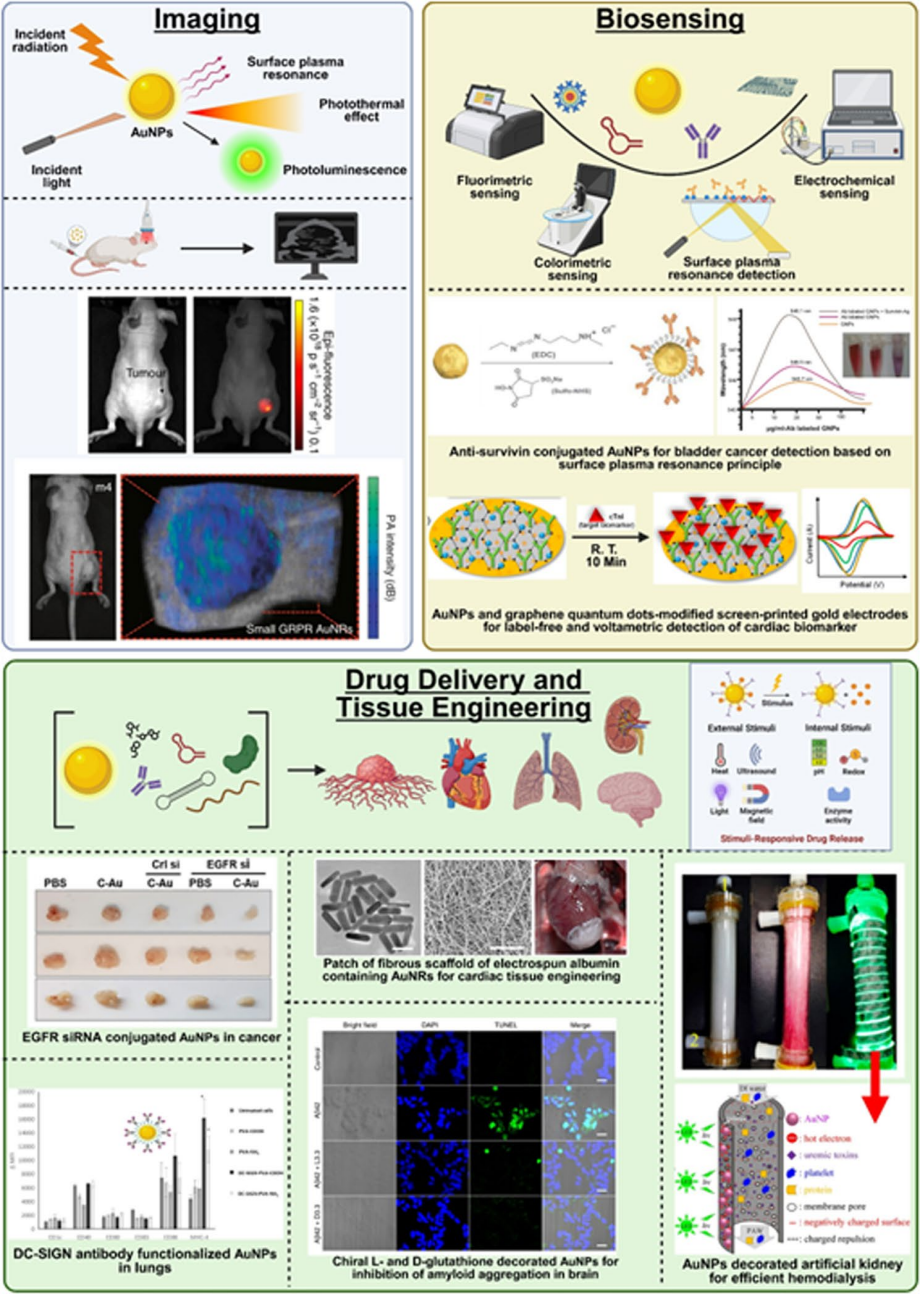
The applications of AuNPs in imaging, biosensing, and drug delivery areas of biomedical field. **A** Illustrates how AuNPs enhance imaging through photothermal effect, photoluminescence, and surface plasmon resonance under incident light for applications like epifluorescence and photoacoustic imaging. Indocyanine green conjugated gold nanorods decorated with stimuli-responsive poly(n-isopropylacrylamide) (PNIPAM) nanogel show five-fold increase in epifluorescence and consistent Signal after 24 h post injection suggesting improved imaging in animal models of prostate cancer [187]. Similarly, photoacoustic imaging of animal model of prostate cancer shows higher and tumor-selective photoacoustic signal upon injecting smaller gold nanorods decorated with G-protein coupled receptor targeting peptide [181, 187]. **B **Shows AuNPs-based sensing platforms fluorimetric, electrochemical, colorimetric, and SPR that enable sensitive detection of disease biomarkers using enhanced signal transduction. Anti-survivin antibody conjugated AuNPs help in highly sensitive and specific detection of surviving protein in the serum and urine samples of bladder cancer patients [214]. The screen printed gold electrode (SPGE) containing AuNPs and graphene quantum dots decorated with anti-cardiac troponin-1 antibodies detect the biomarker of CVDs via electrochemical method within 10 min at room temperature [313]. **C** Depicts functionalized AuNPs carrying therapeutic agents and targeting ligands for site-specific delivery to organs like lungs, heart, brain, and kidneys, improving bioavailability and efficacy. siRNA (directed against epidermal growth factor receptors)-conjugated collagen modified AuNPs (EGFR-si-C-Au) reduced the tumor volume by 60% upon intraperitoneal administration in selective way as compared to various controls in animal models of lung cancer [127]. Similarly, DC-SiGN antibody mediated targeting and activation of immune cells in lungs upon aerosolized delivery of AuNPs [158]. Furthermore, the fibrous scaffold of electrospun albumin containing gold nanorods under NIR radiation help in replacing the infarcted cardiac tissue in suture-free way [147]. Chiral L- and D-glutathione conjugated AuNPs inhibit amyloid-β mediated cytotoxicity and then enantioselectively repairs the memory associated problems in the mouse model of Alzheimer’s disease [168]. Artificial kidney laden with AuNPs under LED illumination help in selective removal of uremic toxins, solutes, and free radicals and pose as an attractive option for hemodialysis in CKD patients [154]. (Create using biorender)

The list of applications of the AuNPs synthesized from physical or chemical or biological methods is non-exhaustive [113, 114]. Well-documented proofs describe their applications in physics, chemistry, biology, electronics, electrical, environmental science, and biomedical fields [113]. Among all domains, the biomedical field remains the most extensively explored area for AuNPs where they have been employed as drug delivery systems, therapeutics agents, and diagnostic agents [114]. In this context, the effectiveness of AuNPs is largely attributed to their ability to engage in key mechanisms such as targeted delivery through ligand functionalization, photothermal conversion via localized heating under NIR light, stimuli-responsive drug release triggered by pH or redox conditions, and contrast enhancement for imaging due to strong optical and X-ray absorption [114, 115]. These mechanisms collectively underpin the multifunctional role of AuNPs in biomedicine: surface ligands enable selective targeting of diseased cells; photothermal properties facilitate minimally invasive tumor ablation; microenvironmental sensitivity allows for controlled drug release; and their electron-dense and plasmonic characteristics make them excellent agents for imaging and biosensing applications [114, 115]. The details of the applications of AuNPs in the fields of (i) drug delivery, (ii) medical imaging, and (iii) biosensing applications are mentioned in subsequent paragraphs (Fig. 3).

### AuNPs for drug delivery

AuNPs-mediated drug delivery has transformed therapeutic strategies for various diseases, offering enhanced permeation, high drug-loading efficiency, protection of delicate drug molecules, and controlled release [116–118]. Table 2 summarizes the different types of AuNPs, their mechanisms, advantages, and limitations in various medical applications. The inert nature of AuNPs contributes to their excellent biocompatibility [114, 119]. Although some toxicological reports suggest that ultrasmall AuNPs (< 10 nm) exert deleterious effects, whereas the larger particles (> 50 nm) show a relatively safe profile. But a large portion of the literature gives firm evidence of their biocompatibility among several in vitro as well as in vivo models [120, 121]. Nanoparticle-mediated drug delivery systems offer several advantages, including enhanced tissue penetration, improved access to poorly vascularized or hypoxic regions within the targeted site (such as the TME), a high surface area-to-volume ratio that allows greater drug loading, and protection of labile drug molecules, such as peptides and proteins from premature degradation or inactivation. Additionally, these systems are highly amenable to surface modifications through various bioconjugation strategies, enabling targeted delivery via ligands like antibodies, improved drug immobilization, and controlled release profiles [116–118]. In addition to these advantages, the AuNPs offer additional merits such as inert nature leading to excellent biocompatibility, elimination from body and no accumulation leading to biosafety, excellent thermal and optical characteristics in order to use them along with external thermal/optical instruments for detection/therapy of a disease, and longer shelf-life owing to improved stability [114, 119]. Many such gold nanoformulations have been shown to be effective for delivering drugs in many diseases. To note among all such diseases, extensive efforts have put towards developing gold nanoformulations against various types of cancer such as breast, lung, colon, oral, prostate, and pancreatic cancer [119].

**Table 2:** An overview of various AuNPs, highlighting their optimal medical applications, underlying mechanisms, benefits, and potential challenges in therapeutic and diagnostic settings

Gold Nanoparticle Type	Investigated Therapeutic and Diagnostic Uses	Mechanism of Action	Advantages	Limitations
Spherical AuNPs (20–50 nm)	Drug delivery, molecular imaging, biosensing	EPR, passive targeting; easy surface modification	Biocompatible, tunable size, multifunctional surface chemistry	Potential for nonspecific biodistribution, aggregation, and renal clearance
Gold Nanorods AuNPs	PTT, cancer theranostics, photoacoustic imaging, osteoporosis therapy	Strong NIR absorption, plasmonic heating for localized ablation, inhibit osteoclastogenesis, promote osteoblast differentiation	Deep tissue penetration; effective tumor targeting via shape-dependent optical properties, bone remodeling potential	Heating can affect healthy tissues; photothermal dose must be carefully controlled, limited skeletal targeting data
Gold Nanoclusters (≤ 5 nm)	Imaging (MRI, fluorescence), neurodegenerative diseases, lung metastasis	High renal clearance; crosses BBB; emits photoluminescence	Low toxicity; suitable for brain imaging and drug delivery	Short circulation time; requires functionalization for stability and targeting
Gold Nanocages (30–60 nm)	Pancreatic cancer theranostics, dual-modal imaging (MRI & FI), targeted drug delivery	Porous structure enables controlled release; optionally functionalized for gene-targeting (e.g., survivin) and dual-modal imaging (MRI/FI)	High specificity, dual imaging (MRI/FI), excellent biocompatibility, renal clearance within 72 h, potential for drug co-delivery	Limited to survivin-expressing tumors; requires surface functionalization; limited payload space due to imaging agents
Gold Nanostars (50–100 nm)	PTT, SERS imaging, cancer diagnostics	Sharp tips enhance local field concentration; strong NIR absorption; tunable plasmon resonance	High photothermal conversion efficiency; sensitive imaging (e.g., SERS); effective tumor ablation	Complex synthesis; shape heterogeneity; potential instability in physiological environments
Gold Nanoshells (100–150 nm)	PTT, atherosclerosis treatment	Core-shell plasmonic resonance; NIR-triggered heating	High photothermal conversion efficiency; minimally invasive	Restricted to surface or shallow tumors due to NIR attenuation in dense tissues; potential RES accumulation
PEGylated AuNPs	Long-circulating delivery, cancer theranostics	PEG coating provides stealth characteristics, reduced RES uptake	Enhanced stability, prolonged half-life, decreased immune clearance	PEG-specific immune responses (anti-PEG antibodies), reduced cellular uptake
Folic Acid-Conjugated AuNPs	Targeted ovarian, breast, and lung cancer therapy	Active targeting via folate receptor-mediated endocytosis	High selectivity for FR-overexpressing tumors; reduced off-target toxicity	Ineffective in FR-negative tumors; possible receptor saturation
Hyaluronic Acid-Coated AuNPs	Colon, breast cancer targeting, anti-inflammatory drug delivery	Binds CD44 and RHAMM receptors; mediates endocytosis in cancer cells	Biocompatible; supports receptor-mediated delivery	Binding efficiency varies with receptor expression; enzymatic degradation risk
Transferrin-Coated AuNPs	Glioblastoma, brain-targeted therapies	Efficient targeting of brain tumors; reduced systemic side effects	Targeted therapy for brain tumors, reduced off-target effects	Limited efficacy in transferrin receptor-negative tumors; potential transferrin competition

In case of colon cancer, several naturally derived anticancer compounds such as resveratrol, known for its antioxidant properties, face the major limitation of low bioavailability, which hinders their therapeutic potential despite their promising efficacy. To improve their bioavailability Kamal et al. used AuNPs and proved better efficacy owing to nanoparticulate delivery in cell and animal models of colon cancer. The authors also labeled their nanoparticles with a radioactive moiety for image guided therapy of colon cancer [122]. Hosseinzadeh et al. synthesized polymer-metal hybrid nanoparticles by combining hyaluronic acid and gold and labeled them with a specific aptamer (MUC1 aptamer) for targeted delivery of nanoparticles. By using light emitting diode and combining photothermal properties of AuNPs, authors proved anti-proliferative properties of the nanoformulation in the cell model of metastatic colon cancer [123]. Similar combination therapy has been used for proving anti-colon cancer potential of AuNPs by other group of researchers [124]. In order to achieve control over release of anticancer drugs and to obtain stimuli responsive nanoformulation, polymers such as chitosan, Poly(lactic-co-glycolic acid), polyurethane have been combined with AuNPs to deliver drugs such as paclitaxel, doxorubicin, and folic acid in cell and animal models of various cancers [125].

Lung cancer is another type of cancer that is characterized by the highest mortality rate against which several gold nanoformulations have been developed. For example, Thambiraj et al. have developed docetaxel loaded AuNPs to combat the cell model of squamous cell carcinoma [126]. In non-small cell lung carcinoma cell models (A549 cell line), Yu et al. showed delivery of siRNA against epidermal growth factor receptors via collagen coated AuNPs and proved their efficacy in terms of tumor size reduction properties in cell as well as animal models [127]. Similar reports have been published for other SiRNAs directed against apoptosis protein 2 inhibitor and specificity protein 1 that were delivered through AuNPs [128, 129].

AuNPs have also shown immense potential against pancreatic neuroendocrine tumors (PNETs) and thyroid cancer as well. One such notable example is CYT-21,625 [130] first-in-class gold nanomedicine designed to target tumor vasculature and cancer cells by specifically delivering recombinant human tumor necrosis factor alpha (rhTNF-α) and a paclitaxel prodrug analog (JZ-21625) [130]. This gold nanomedicine is designed to target tumor vasculature and cancer cells by delivering rhTNF-α and a paclitaxel prodrug. Testing on mice with metastatic thyroid cancer and insulin-secreting PNETs demonstrated the effectiveness of CYT-21,625 without observed toxicities. These promising results suggest the potential for CYT-21,625 to be further investigated in clinical trials for patients with advanced PNETs and thyroid cancer [130]. Mechanistically, rhTNF-α binds to its receptors on tumor neovasculature, inducing apoptosis of vascular endothelial cells and destroying tumor blood vessels. Upon release in the TME, CYT-21,625 releases native paclitaxel, exerting a direct antitumor effect. Thus, the immunomodulatory coatings mitigate adverse immune reactions, improving biocompatibility and systemic circulation [131]. By precisely controlling drug release and enhancing tumor penetration, these innovative formulations promise to overcome challenges associated with conventional chemotherapy and advance personalized cancer therapy strategies. Another example is, CytImmune utilizes AuNPs coated with tumor necrosis factor-alpha (TNF-α), which selectively targets and kills endothelial cells in tumor blood vessels [132]. CYT-6091, CytImmune’s gold-plus-TNF-α drug, has shown promising safety and tolerance in phase 1 trials. In addition to CYT-6091, there are other notable examples of AuNPs-based controlled drug delivery, where researchers have developed AuNPs-drug conjugated with other chemotherapeutic drugs such as paclitaxel, doxorubicin and cisplatin, thereby reducing off-target effects and improving treatment outcomes in cancer patients [132].

Breast cancer, characterized by the highest incidence rate across the globe, is one type of cancer against which several AuNPs have been developed. For example, Pechyen et al., used green AuNPs prepared from Anacardiaceae peel extract and showed their dose-dependent activity in breast cancer cell line. Authors proved that the action of AuNPs having biological corona of *Anacardiaceae* flavonoids and phenols was more potent as compared to extract due to small size of nanoparticles, their enhanced penetration into the cells, and exerting oxidative stress to the cells [133]. Liu et al. exploited the enhanced permeation and retention properties of nanoparticles for combination treatment of breast cancer and lung metastasis. Authors reported paclitaxel loaded gold nanoclusters prepared using hyaluronic acid nanoparticles and showed that at optimal size of 100 nm, they enter into the breast tumor and owing to the action of hyaluronidase enzyme, 100 nm particles breaks down to release 20 nm sized gold nanoclusters. These nanoclusters, along with loaded paclitaxel, impart cytotoxic action in cell and animal models of breast cancer [134]. With similar principle, Cun et al. used a size-shrinkable gelatin nanoparticle loaded with doxorubicin bearing AuNPs in different breast cancer cell line under the influence of matrix metalloproteinase enzyme [135]. Apart from breast cancer, this strategy has also been used to prove the efficacy of AuNPs against melanoma and brain cancer (glioma) too [136, 137]. AuNPs have also been combined with novel immunotherapy, where Emami et al. has conjugated Cetuximab to gold nanorods and proved elevated efficacy against triple negative breast cancer models [138].

Wang et al. have explored action of green AuNPs in androgen-resistant prostate cancer. Authors used paclitaxel loaded nanoparticles of poly(ethylene oxide)(PEO)-poly(propylene oxide) (PPO) copolymer to incubate with chloroauric acid for in situ synthesis of AuNPs. They showed the efficacy of these polymer-gold hybrid nanoparticles in cell and animal models of prostate cancer in terms of inhibiting calcium ion channel, enhancing cell cycle arrest, elevating temperature, and generating reactive oxygen species (ROS) leading to potent cytotoxicity [139]. With similar approach Zhang et al. provided evidence of anticancer activity of double stranded DNA conjugated AuNPs at metabolic level in prostate cancer cells with specific alterations in the glycolysis and lipid metabolic pathways [140]. On the other hand, Wang et al. employed prostate-specific membrane antigen 1 ligand decorated ultrasmall AuNPs (having size 3–6 nm) along with a contrast agent for image guided therapy of prostate cancer. Authors proved high biocompatibility, preferential targeting, and elevated tumor distribution of their nanoparticles [141]. AuNPs also showed diagnostic potential in adrenal cancer. A notable example is biofunctionalized AuNPs developed by Godoy et al. for the detection of normetanephrine, which is an important biomarker in the diagnosis of adrenal tumors such as pheochromocytoma or paraganglioma [142]. They functionalized the AuNPs with two different groups, benzaldehyde-terminated ligand and N-acetyl-cysteine, for the recognition of amino alcohol moiety and phenolic hydroxyl group on normetanephrine. This led to interparticle-crosslinking aggregation resulting in a change in the color of the solution from red to blue based on normetanephrine concentration.

Apart from cancer, the AuNPs are also useful in the treatment of cardiovascular diseases (CVD) [143]. In an attempt to cure the CVD and CVD associated complications, the tissue engineering principles and various scaffolds have shown great potential. Cardiac tissue engineering is itself a growing field in which the AuNPs can be effectively used owing to their biocompatibility as well as their excellent electrical properties [144–146]. For example, in two separate reports, Tal Dvir et al. and Shevach et al. have shown functional repair of damaged heart muscle using polymeric scaffold embedded with two different types of gold nanomaterials. Tal Dvir et al. used chemically synthesized anisotropic gold nanowires with alginate scaffold [144]; whereas, Shevach et al. used AuNPs with polycaprolactone–gelatin mixed fibrous scaffold [145]. Both groups showed that owing to high electrical conductivity of gold nanomaterials (nanowires and nanoparticles), the originally poorly conductive polymeric scaffold becomes excellent electrical conduit for cardiomyocytes. In another similar report, You et al. showed that the AuNPs decorated polymeric scaffold showed improved expression of Connexin-43 protein that is essential for ventricular conduction velocity. Authors showed that such AuNPs loaded thiol functionalized hydroxyethyl methacrylate scaffold show excellent electrical conductivity and biocompatibility leading to improved adhesion of cardiomyocytes and increased expression of Connexin-43 upon electrical stimulation. Such elevated protein expression further helps regulation of the cell–cell communication, electrical coupling of cells, and their contractile behavior [146]. With similar concept, Malki et al. used excellent thermal conductivity of the AuNPs to produce a fibrous patch of albumin and showed that with local heating using IR laser, the AuNPs help in integration of fibrous albumin scaffold with local cardiac tissue negating the need of sutures; thus produced suture-free scaffold for use in cardiac complications [147]. Somasuntharam et al. used specific DNAzyme against TNF-α to reduce myocardial infarction associated inflammation. Authors showed that upon DNAzyme loading the AuNPs attained hydrodynamic size of around 80 nm and owing to this size, the nanoparticles were successfully internalized better than lipofectamine mediated delivery of DNAzyme [148].

Contrary to these reports, Abdelhalim has shown that the exposure of the small AuNPs (< 50 nm) may impart cardiac tissue toxicity in terms of presence of dilated blood vessels, extravasations of red blood cells, muscle hyalinosis, disturbed muscle fascicles, and presence of the inflammatory cells [149]. The author did not find any cardiotoxicity with AuNPs having average size of 50 nm suggesting that the nanotoxicity is dependent on the particle size. One of the reasons for such toxicity could be interaction of small AuNPs with the ion channels leading to their irreversible blockage as shown by Leifert et al. [150]. Irrespective of these reports, in 2017, Kharlamov et al. conducted first-in-human trials for AuNPs mediated PTT of atherosclerosis and showed that it exhibited improved results in terms of biosafety, improved patient survival, reduced adverse reactions, and reduced incidence of thrombosis and atherosclerosis post treatment as compared to commercial drug delivering stent [151]. In a different report, Spivak et al. used levosimendan loaded AuNPs for the treatment of heart failure under the influence of ultrasound waves for improved penetration. Although authors stated that no significant beneficial effect was observed in the recovery of animal models, but owing to significant penetration of AuNPs under ultrasound source, the drug was delivered in the cytosol of cardiomyocytes leading to improved life longevity [152].

The AuNPs have also found their way into the diagnosis and treatment of chronic kidney diseases (CKDs) [153]. Patients with chronic renal failure are often recommended to follow hemodialysis procedure which has serious drawbacks such as dialysis-induced oxidative stress, non-specific protein adsorption, platelet adhesion, and activation of coagulation cascade followed by chronic inflammation. Hence, to circumvent these drawbacks, Chien-Chen et al. developed an artificial kidney containing AuNPs. Upon illumination of this AuNPs laden artificial kidney using green light emitting diode during dialysis, authors observed reduced platelet adhesion, reduced non-specific protein adsorption, reduced coagulation along with efficient removal of urine toxins. This report has widened the applicability of AuNPs in conjunction with artificial organs [154].

In case of respiratory diseases, AuNPs can be effectively used via nebulization technique or intranasal injection technique [155–157]. For example, Barreto et al. employed the antioxidant and anti-inflammatory properties of AuNPs in animal models of allergen inducing asthma. They reported that the AuNPs treatment significantly alleviated the pathological changes of allergen induced asthma and inhibited airway hyperreactivity, eosinophil infiltration, and peribronchiolar fibrosis while reducing excessive mucus production [156]. To address the question of nanotoxicity of AuNPs when administered with nebulization technique, Urso et al. have performed in-depth study of biodistribution of AuNPs, their clearance, and their effects on lung anatomy and physiology. Authors showed that upon nebulization, the AuNPs get distributed bilaterally and uniformly. They also observed no effect on the total lung volume and no sign of inflammation or tissue damage that confirmed their biocompatibility and biosafety [157]. In the 3D model of lung cells, aerosolized delivery of DC-SIGN antibody functionalized AuNPs resulted in very specific targeting of macrophages and dendritic cells that ultimately led to reduced immune response against allergens. Such immunoengineering approaches have opened new doors for the treatment of severe inflammation which occurs in chronic obstructive pulmonary diseases [158].

Owing to their antioxidant as well as anti-inflammatory properties, AuNPs have shown promising effects against several inflammatory conditions such as rheumatoid arthritis, uveitis [159]. Pareira et al. used chemically synthesized 30 nm gold nanospheres and tested their activity against uveitis. Authors found that treatment with AuNPs reduced the levels of TNF-α, and myeloperoxidase in aqueous humor along with significant reduction in oxidative damage [160]. Apart from these applications, Dave et al. recently showed that the folic acid decorated AuNPs can be used to deliver sorafenib tosylate to treat diabetic retinopathy. Authors used green AuNPs synthesized from ginger extract and showed sustained release of sorafenib as well as subsequent prevention of neovascularization that is common in diabetic retinopathy [161]. Recently Rafik et al. have used methotrexate conjugated AuNPs in animal models for the treatment of rheumatoid vascular dysfunction, which is a common symptom in rheumatoid arthritis patients having cardiovascular complications. Authors showed that owing to immunomodulatory action of methotrexate and anti-atherosclerosis action of AuNPs, the animal models of rheumatoid vascular dysfunction showed improved physiology in terms of reduction of inflammation and inflammatory makers as well as improved lipid profile [162]. The action of AuNPs against rheumatoid arthritis and associated complications was found to be size dependent. Abdel-Hakem et al. showed that the AuNPs of size 25 nm showed excellent therapeutic efficiency in collagen induced arthritis animal models and exhibited reduction in the swelling of soft tissue as well as stifle joints, associated erosions, ankylosis, and osteopenia upon treatment [163]. James et al. also compared action of gold salts (aurothiomalate, aurothioglucose, auranofin) and AuNPs against rheumatoid arthritis and proved their biocompatibility with macrophages while exerting better and potent anti-arthritic action as compared to that of salts [164].

In case of neurodegenerative diseases such as Alzheimer’s diseases AuNPs have shown promising therapeutic potential with their anti-aggregation and anti-amyloidogenic properties [165–167]. Simulation as well as experimental evidence is now available that shows beneficial effects of chiral AuNPs against memory related impairment of Alzheimer’s disease [168]. Apart from their action against pathogenic protein aggregation, the electrochemically synthesized AuNPs with organic coatings have proven to be useful in improving the bioenergetics as well as in the neuroprotection by mimicking NADH dehydrogenase enzyme role [169]. Via conjugation with a peptide ligand of transferrin receptors, the AuNPs have shown to cross the toughest BBB via receptor mediated endocytosis process to interact with amyloid fibrils. This approach has opened new avenues for delivery of anti-aggregation and anti-amyloidogenic therapeutic molecules across BBB via AuNPs [170]. In an interesting report, Gao et al. have shown that gold nanoclusters having size greater than AuNPs accelerate the aggregation of β-amyloid which proved that the size of the AuNPs also play important role in their therapeutic action against neurodegenerative diseases [171]. On the other hand, Moore et al. proved the beneficial effect of surface coatings on the AuNPs in their action against β-amyloid protein aggregation [172].

### AuNPs for medical imaging 

In modern times, because of the availability of advanced techniques, the field of medical imaging has seen significant progress in early detection of anatomical and physiological anomalies in early stages [173, 174]. This helps in taking precautionary measures in order to avoid disease progression and thereby worsening patient’s condition. At present, many techniques such as magnetic resonance imaging (MRI), computer tomography (CT), positron emission tomography (PET), optical coherent tomography (OCT), photoacoustic imaging, and X-ray imaging are available for detection of wide array of diseases [173, 174]. However, the availability of only imaging techniques does not guarantee clear imaging and detection. In order to enhance imaging, different types of contrast agents are employed which improve signal to noise ratio yielding accurate imaging [173]. As described earlier, the AuNPs have unique optical properties similar to other inorganic particles. The AuNPs show excellent attenuation of X-rays as denoted by higher Hounsfield unit values which makes them ideal for CT imaging as compared to conventional iodine based agents [173, 175]. Furthermore, better photothermal energy conversion properties of AuNPs make them suitable for photoacoustic imaging. Owing to SPR effect which comes from oscillation of free electrons on the surfaces of AuNPs and stronger back scattering at higher wavelengths (> 800 nm), they can be used effectively in OCT imaging [176]. It has been shown that the addition of a radiolabeled moiety to AuNPs proved better in PET and SPECT imaging owing to the improved contrast. AuNPs offered improved radio-stability in vivo that helped in better PET imaging [177, 178]. Also, by combining already available contrast agent with AuNPs via bioconjugation or capping or adsorption methods, dual advantages of AuNPs and contrast agents can be merged to yield synergistic effect in terms of improving image quality by several folds [173, 175].

The very first use of bare AuNPs in X-ray imaging dates back to 2004, when Hainfeld et al. used them for imaging the vasculature and organs of mice [179]. Recent literature suggests tremendous applicability of AuNPs as contrast agent to improve medical imaging in diseases like cancer [180]. Chen et al. have shown that miniature gold nanorods (8 × 49 nm) having size significantly smaller than conventional gold nanorods (18 × 120 nm) can be used for improving the contrast of biological samples in the second NIR optical Widow. Authors stated that in this window, very few contrast agent show applicability Limiting use of this Window; however, the miniature gold nanorods solve this problem owing to their 3 -fold higher thermal stability and 3.5-fold stronger photoacoustic signal [181]. Duan et al. also developed a cluster of polydopamine stabilized AuNPs that had great stability and intense absorption in the Wide range of 400–1300 nm along with photothermal efficiency going beyond 80% in both first as well as second NIR Window that would allow better imaging of tumors in vivo. Authors attributed this improvement in imaging to the intraparticle plasmonic coupling among the particles Situated in close proximity within the nanocluster of size 50 nm [182]. The evidence is also available that states that the shapes of the gold nanoformulation have Significant impact on their contrast properties. Gao et al. used gold nanospheres, gold nanocubes, gold nanotriangles, gold nanorods, and gold nanobranches to study their contrast properties and found that the gold nanobranches exhibited strong two-photon photoluminescence properties that was around 50,000 times higher than gold nanosphere [183]. Tang et al. have shown use of AuNPs in optical coherence tomography. The authors developed gold nanoprisms having polyaniline shell on the surface that exhibited pH responsive properties and exhibited different absorption properties in acidic and alkaline pH conditions. Authors have predicted its application in imaging the interior of tumor by validating this OCT contrast nanoformulation in gelatin based phantom formulation that was used as tumor mimic with acidic core and neutral pH shell. Owing to the reversible dielectric function of polyaniline shell, the surface plasma resonance properties of gold nanoprism can be modulated due to which the pH dependent OCT imaging can be done with the response time of less than a second [184]. Wi et al. prepared novel disc shaped gold nanoformulations and employed them in OCT imaging [185]. In case of gold nanodiscs, the authors attributed excellent contrasting properties in OCT imaging to their 2D shape. Such shape made them amenable to the tuning the ratio of light absorption to the scattering as well as to the responsiveness to incident light. Authors also stated that the stacked discs gave even better performance in OCT imaging owing to the combination of optical properties of two nanodiscs upon increase in the overall geometry. Using a phantom tissue authors have proved its utility [185]. Such different shapes of AuNPs can prove to be useful in the imaging of tumors. These reports also state the fact that not just the size but also shape of AuNPs plays a vital role in their optical properties. As AuNPs possess anti-photobleaching property, they are ideal agents for the photoacoustic imaging [186]. For example, Chen et al. used nanoconstructs made of poly(n-isopropylacrylamide) nanogels and gold nanorods which was prepared by in Situ seed mediated growth method. In the animal model of prostate cancer, upon intravenous injection, these nanoconstructs were shown to enhance the contrast by 30-fold as compared to AuNPs alone [187]. In a different report, Rabee Cheheltani et al. prepared poly-di(carboxylatophenoxy)phosphazene polymer coated gold nanospheres. The coating of biodegradable polymer on AuNPs with 5.5 nm size helped in improving biocompatibility of Au-polymer nanoformulations.

Furthermore, encapsulation of many ultrasmall AuNPs in polymeric matrix also improved the photoacoustic contrast and thereby the CT imaging of animal models. Also, upon the slow degradation of biodegradable and biocompatible polymer matrix, the released ultrasmall-AuNPs were completely and slowly eliminated without signs of nephrotoxicity in animal models [188]. Some gold nanoformulations have been used for “theranostic” purposes. The word theranostic suggest ability of agent/system to exert therapeutic and diagnostic properties once administered [189]. Such theranostic ability of AuNPs gives additional advantages especially in non-communicable diseases like cancer where the tumor can be targeted using imaging system and then treated with a therapeutic agent. For example, Ming Chen et al. used metal organic frameworks (MOFs) having surface decoration of gold nanoshells for multimodal imaging guided PTT of in vitro and in vivo models of breast cancer. Authors used NIR laser to irradiate the MOFs loaded with gold nanoshells and upon irradiation, they showed that the MOFs-gold nanoshells converted photothermal energy with the efficiency of 74% yielding brighter and better output in photoacoustic imaging. Authors also showed that the octahedron shaped MOFs-gold nanoshell had higher Hounsfield unit values as compared to standard iohexol and showed higher X-ray attenuation. With such higher Hounsfield unit values, the MOFs-gold nanoshell showed high contrast power and improved CT assisted imaging. This enabled the authors to perform image guided therapy of breast cancer. Using IR camera, the authors monitored the tumor volume in control (without nanoformulations and NIR laser irradiation) and treatment groups (with nanoformulations and with NIR laser irradiation) and showed significant tumor reduction in animals upon MOFs-gold nanoshell treatment under NIR laser irradiation without any observable damage to other organs [190]. In another report, Yu Fan et al. used nanohybrids made of dendrimers loaded with AuNPs and gadolinium (III) and then targeted hypoxia of TME. Authors showed that the gold core of 3.2 nm in the dendrimer showed excellent attenuation of X-rays resulting in imaging with excellent contrast. Additional contrast improvement was also provided by the encapsulated gadolinium (III) compound. They also proved that their nanohybrids had excellent penetration ability and upon X-ray irradiation the nanohybrids produced ROS leading to DNA damage, prevented DNA repair and led to tumor cell death. Additionally, the hypoxia targeting moiety (Nitroimidazole) loaded onto these nanohybrids helped in the sensitization of tumor cells in vitro and in vivo [191].

In similar lines of research, Taoxia et al. used photoacoustic imaging guided chemotherapy using hydrophilic anticancer drugs co-encapsulated with AuNPs inside the β-cyclodextrin nanocomposites to treat animal models bearing 4T1 cell tumors. Authors showed that with increase in AuNPs nanocomposite concentration, the photoacoustic Signal increased Linearly. The mice treated with these nanocomposites showed better results in terms of tumor size reduction upon intravenous treatment with nanocomposites and then irradiation with 808 laser for 10 min [192]. Golsanamlu et al. have shown that the cube shaped nanocomplexes of polydopamine functionalized AuNPs and magnetic ferric chloride nanoparticles conjugated via hyaluronic acid polymer using carbodiimide chemistry bind specifically to CD44 receptors for initiating receptor mediated endocytosis process and upon binding they emit strong fluorescence signal allowing fluorescence imaging of breast cancer cells [193]. Spherical AuNPs are known for their well-defined plasmon resonance peak, making them ideal for applications in imaging and sensing. For instance, Abdelkader et al. recently showed the neuroprotective effect of spherical AuNPs against radiation-induced brain damage in rats. The study concluded that AuNPs with alpha-lipoic acid mixture represent a potential candidate for alleviating radiation-associated brain toxicity [194]. Gold nanorods, with their anisotropic shape, exhibit tunable plasmon resonance, large enhancement of the local electromagnetic field near the two tips, and intrinsic geometrical anisotropy, making them ideal candidates for PTT in cancer treatment [195, 196]. Similarly, with their sharp protrusions, gold nanostars enhance the surface area for biomolecule attachment, enabling sensitive detection in biosensing applications [197, 198]. Anisotropic shapes such as nanorods or nanostars introduce directional properties that are particularly advantageous in PTT, where selective light absorption induces localized heating, targeting specific cells or tissues [199]. These findings demonstrated that the absorption of NIR light by the gold nanorods resulted in efficient conversion to heat, leading to selective destruction of cancer cells while sparing surrounding healthy tissue. This research highlighted the potential of AuNPs with anisotropic shapes for precise and localized cancer therapy [200]. Another recent study showed that daily administration of a suspension containing gold nanocrystals reversed deficits in brain energy-related metabolites and led to notable functional improvements in patients with multiple sclerosis (MS) and Parkinson’s disease (PD) [201].

The AuNPs have also shown their utility in imaging of cardiac tissue, vasculature, and renal tissue [173]. Kee and Danila et al. used collagen-binding adhesion protein 35 (CNA35) functionalized AuNPs for vascular imaging while specifically targeting the collagen of myocardial scar. Authors showed that these nanoparticles owing to their longer persistence in the blood as compared to conventional iodinated blood pool agents allow CT imaging of vasculature. Additionally, owing to the CNA35, authors could perform imaging of collagen deposition in myocardial scar. Their overall results suggested that CT imaging of cardiac tissue and vasculature was improved owing to the AuNPs [202]. The AuNPs conjugated with anti-collagen antibodies have improved the imaging of fibrotic kidneys owing to the improved retention of antibody labelled AuNPs [203]. Yu et al. synthesized NIR emitting glutathione decorated AuNPs for non-invasive imaging of kidneys and demonstrated capability of AuNPs in reporting the kidney dysfunction state in unilateral obstructive nephropathy that cannot be diagnosed with usual kidney biomarkers [204]. Non-invasiveness, cheaper cost, and reliable robust results makes AuNPs an attractive agent in diagnosis via imaging. In conclusion, the AuNPs with or without contrast agents can assist in currently available medical imaging platforms and can elevate their efficiency by multiple orders of magnitude.

### AuNPs for biosensing

According to classical definition, the biosensing is the process of monitoring (qualitative way) or measuring (quantitative way) a chemical or biological or biochemical reaction via generating signal (colorimetric, fluorimetric, luminescent etc.) that is in proportion with the progress of reaction [205]. Owing to the special optical properties of several types of AuNPs as well as due to their capability to undergo bioconjugation with various dyes or fluorophores, they have been used successfully as biosensors. Starting from simple aqueous suspension diluted in reaction buffer to recently introduced complex, wearable, and handy point-of-care diagnostic devices, the AuNPs have successfully shown their potential in biosensing applications [206]. Till date, several types of gold nanoformulations such as gold nanospheres, gold nanoclusters, gold nanowires, gold nanocages, and gold nanorods have been used for biosensing purposes [207, 208]. One notable example of gold-based diagnostics is the United States Food and Drug Administration (US-FDA) approved Verigene detector developed by Nanosphere [209]. This innovative device utilizes AuNPs coated with oligonucleotides to capture and identify genetic sequences associated with infectious agents. Verigene detectors can rapidly detect various infectious pathogens, which expedite the detection process, with results available within 2–3 h compared to traditional techniques, which often require 2–3 days to diagnose an infectious pathogen in a patient’s sample. This enables healthcare providers to promptly administer targeted antibiotics, minimizing unnecessary broad-spectrum antibiotic use and mitigating the risk of antimicrobial resistance. Especially in diseases like cancer, the biosensors are of utmost importance because they can provide accurate and early diagnosis that can help in deciding the treatment direction for the patients. Several reports are available that show use of gold-based biosensors for detection of cancer biomarkers. For example, Xiao et al. used AuNPs decorated bismuth selenide nanosheets for colorimetric detection of α-fetoprotein and prostate-specific antigen from real-life clinical samples at the picomolar levels [210]. Similarly, Rajamanikandan et al. used β-cyclodextrin decorated AuNPs for specific determination of cysteine; a classical biomarker for many medical conditions, from clinical samples of serum and urine [211]. Cui et al. developed a AuNPs based biosensor for detecting the femtomolar quantities of microRNA-21 which is a well-known biomarker of breast cancer. Using principle of electrogenerated chemiluminescence (ECL) biosensing method, authors generated bridge DNA and AuNPs composites. Authors showed that with such highly sensitive biosensor, the Limit of detection reached to the level of 3.2 × 10^−18^ M which was far better than state-of-the-art ECL based biosensors [212].

In another recent research, anti-HER2 antibodies functionalized dendrimers encapsulating AuNPs and gadolinium-based contrast agent have been developed to target HER2-positive cancer cells. By utilizing the high affinity of anti-HER2 antibodies for HER2 receptors, these AuNPs offered a promising strategy for selective targeting of HER2-positive cancer cells. Moreover, by employing high contrast power of AuNPs and gadolinium-based contrast agent, the authors showed applicability of this AuNPs-dendrimer nanoformulation in the image guided therapy of breast cancer [213]. Jazayeri et al. used anti-survivin antibody conjugated AuNPs for detection of survivin protein in bladder cancer patients with the sensitivity and specificity of detection above 90% [214]. The molecular dynamic simulation and experimental study done by Mahani et al. revealed that chemically synthesized citrate-stabilized AuNPs decorated with anti-prostate specific antigen antibody can be used to detect the antigen in prostate cancer patients at the level of 0.2 ng/ml with specific interactions and binding of nanoparticle-bound antibodies and the antigen [215]. Similar to antibodies, the peptide aptamers against specific oncoproteins like Mdm2 which is overexpressed in leukemia can be conjugated on the AuNPs. Retout et al. gave proof of concept for such “Mdm2 binding peptide aptamer” conjugated AuNPs biosensor and showed that the sensitivity can go to nanomolar range [216].

In CVDs, AuNPs have also shown tremendous potential in biosensing the early markers. Byzova et al. developed a multiplexed detection system for sensing various cardiac and inflammatory markers, such as myoglobin, D-dimer, and C-reactive protein etc. that are generally assessed to predict risk factors post myocardial infarction [217]. Mansuriya et al. combined antibody-labelled AuNPs with graphene quantum dots to synthesize an immunosensor to detect femtogram-level quantities (limit of detection (LOD) ~ 100 fg/ml) of cardiac troponin-I, a known biomarker for the early diagnosis of acute myocardial infarction [218]. Similar femtogram level detection of C-reactive protein mediated by anti-C-reactive protein antibodies labelled gold nanowires has been validated by Vilian et al. [219].

In CKDs also, AuNPs have been used for biosensing purposes [153]. Marom et al. used ultrasmall spherical AuNPs of size 3–4 nm stabilized by various organic Ligands such as 2-ethylhexanethiol, tert-dodecanethiol, hexanethiol, and dibutyl disulfide and immobilized them on solid substrate like silicon wafer and they attached this assembly to gas chromatography machine for the breath analysis of CKD patients. From breath analysis, the authors identified several substances released from biochemical processes and accumulated toxins after the loss of kidney function. It was directly correlated with the grade of CKD [220]. Shaikh et al. used polymer-gold nanocrystal composite (polyaniline-gold nanocrystal composite) on which specific antibodies against albumin were immobilized for detecting early stage CKD [221]. With similar concepts, several other AuNPs based biosensors based on colorimetric or fluorimetric or luminescence detection have been developed for detection of protein biomarkers such as apolipoprotein A1, Her2, thyroglobulin, osteocalcin and ferritin etc. in various types of cancers [222, 223] Autoantibodies generated against IgG in arthritis patients; also known as rheumatoid factors are one of the excellent biomarkers for early detection of rheumatoid arthritis. Veigas et al. used human IgG-Fc conjugated AuNPs to detect rheumatoid factor in the serum by employing their aggregation based colorimetric properties [224]. Based on similar principle, very recently, Middle East respiratory syndrome coronavirus (MERS-CoV) detection has also been achieved using double-stranded DNA self-assembly shielded AuNPs by Kim et al. [225]. In another recent study, Marin et al. developed a biosensor based on AuNPs functionalized with DNA aptamers for the detection of *Staphylococcus aureus* [226]. Similarly, AuNPs have been incorporated into lateral flow assays to enable rapid and sensitive detection of the influenza virus [227, 228]. In this approach, AuNPs functionalized with anti-influenza antibodies were immobilized on the test Line of the lateral flow strip. Upon exposure to influenza virus antigens in clinical samples, the AuNPs generated visible Lines, indicating a positive result within 15 min, with a sensitivity comparable to that of PCR-based methods [228].

Overall, we can say that the AuNPs can be employed for drug delivery, therapy, diagnosis, imaging, and theragnosis purposes. While exerting any of these actions, the final output (in terms of therapeutic effect or contrast level in imaging or biosensing capability) is always affected by the size, shape, and surface chemistry of the AuNPs.

## Pharmacokinetics of AuNPs

### Systemic distribution and tumor accumulation

 In the context of cancer therapy, AuNPs are generally believed to accumulate in tumor tissues due to the leaky vasculature and inefficient lymphatic drainage, a phenomenon known as the EPR effect. Most pharmacokinetic studies to date have focused on intravenous (IV) administration of AuNPs, given its ability to ensure rapid and widespread systemic distribution. However, recent reports, both at the laboratory level [229] as well as clinical trial levels [230], suggest that EPR effect is not the only way for endocytosis and accumulation of AuNPs in the tumor cells. While traditionally accepted, recent studies suggest that transcytosis, an active vesicle-mediated transcellular transport mechanism, also plays a significant role in AuNPs extravasation. Unlike passive diffusion through endothelial gaps, transcytosis involves receptor-mediated uptake, intracellular trafficking, and exocytosis [231]. This route is regulated by factors like pH and transporter expression, and has been shown to influence AuNPs bioavailability, systemic retention, and tissue targeting in both tumors and renal tissues [229, 232]. In some cases, both routes contribute to tumor entry, but active transport has been shown to dominate over the classical passive EPR mechanism [229]. For instance, PEGylated AuNPs of 15, 50, and 100 nm have revealed a predominant reliance on transcellular transport, with up to 97% of particles traversing endothelial barriers via vesicle-mediated processes rather than paracellular gaps. This active route, involving only a small subset (~ 21%) of endothelial cells, underscores the heterogeneous nature of tumor vasculature. Notably, nanoparticle size plays a critical role: smaller AuNPs extravasate more readily but show reduced retention, whereas larger particles face higher entry resistance yet persist within the TME [233, 234].

Recent advances in AuNPs delivery have introduced alternatives to IV administration, improving systemic distribution. Oral formulations, in particular, have shown promise in treating arthritis and neurodegenerative disorders, with good tolerability and minimal gastrointestinal side effects. For instance, oral delivery of CEA-functionalized AuNPs using enteric-coated capsules has shown successful systemic absorption in mice, with consistent accumulation in liver and spleen tissues and localized immune activation without systemic toxicity. Authors showed the ability of orally administered AuNPs to cross intestinal barriers, likely via active transcytosis mechanisms, and reach systemic circulation in a biologically functional form [235]. Bhagat et al. 2025 developed pH-responsive alginate-based microcapsules to protect AuNPs from gastric degradation and facilitate targeted release in the small intestine. Upon oral administration in rats, these microcapsules enabled significantly higher blood absorption of AuNPs compared to unencapsulated controls, with no signs of organ toxicity, underscoring their potential for safe and effective systemic delivery via the oral route [237].

In addition to oral and cell-assisted approaches, intradermal (ID) and intramuscular (IM) injection of AuNPs represents another promising route for achieving systemic exposure while avoiding the rapid clearance often seen with IV delivery. When administered into muscle tissue, AuNPs can slowly diffuse into nearby capillaries, creating a reservoir effect that supports gradual absorption into the bloodstream. This extended release profile is influenced by particle characteristics such as size and surface coating, as well as the vascularity of the injection site. Smaller nanoparticles tend to disperse more quickly, whereas larger ones remain localized for longer periods, allowing for a more controlled pharmacokinetic profile [238]. IM delivery also avoids first-pass liver metabolism and may reduce immediate immune recognition, making it a favorable option for applications like depot-based therapies or nanoparticle-enhanced vaccines. For instance, Coelho et al. demonstrated that subcutaneously injected AuNPs conjugated with ovalbumin (AuNPs@OVA) improved wound healing in mice, especially when preceded by oral exposure to the same antigen. These particles, averaging ~ 6.8 nm in diameter, induced systemic immunological shifts and tissue remodeling, outperforming conventional alum-based adjuvants in both antibody generation and repair outcomes [239]. Similarly, Hanna et al. 2023 showed that intradermal injection of AuNPs loaded with a diabetes-associated autoantigen recruited clonally expanded, antigen-specific T cells to the injection site in humans. These immune cells remained in situ for months and exhibited memory and cytotoxic phenotypes, indicating the potential of intradermal AuNPs as a platform for targeted immune modulation and long-term antigen tracking in therapeutic vaccination strategies [240]. While not as extensively explored as IV routes, early evidence suggests that IM and ID administration of AuNPs can provide safe, sustained systemic delivery with minimal early toxicity. Together, these alternative approaches hold promise for improving biodistribution, prolonging circulation, and enhancing therapeutic efficacy of AuNPs-based formulations.

### Influence of physicochemical properties

The physicochemical properties of AuNPs-particularly shape, size, and surface charge, play a vital role in governing their biological interactions, therapeutic efficacy and fate. Shape, for instance, significantly affects both biodistribution and cellular internalization. Mitchell et al. (2020) reported that spherical and larger nanoparticles tend to adhere more readily to vascular walls, whereas rod-shaped AuNPs traverse capillaries more efficiently and penetrate tissues more effectively, often facilitated by exosome-mediated transport [116]. In contrast, Xie et al. (2017) demonstrated different findings in RAW264.7 cells using methylpolyethylene glycol-coated anisotropic AuNPs in star, rod, and triangular shapes. Their study revealed that the highest cellular uptake was seen with triangular AuNPs, followed by rods, with stars showing the lowest uptake efficiency [241]. This suggests that nanoparticle shape not only influences tissue adhesion and vascular traversal but also plays a crucial role in determining the internalization pathways in cells. Another study by Yui et al. 2017, demonstrated that larger AuNPs, specifically 50 nm spheres and 40 nm stars, delivered siRNA more effectively in U87 glioblastoma cells than smaller 13 nm spheres, due to enhanced uptake and improved endosomal escape [242].

Surface charge is equally important in modulating circulation time, immune recognition, and tumor accumulation. Positively charged or uncoated AuNPs are rapidly cleared by the MPS, whereas neutrally or slightly negatively charged particles exhibit extended systemic circulation and improved tumor penetration. For instance, Peter et al. (2018) demonstrated enhanced cellular uptake and therapeutic response with positively charged AuNPs in cancer models [243]. Conversely, other studies have shown that negatively charged particles maintain longer circulation times and preferentially accumulate at tumor sites, improving imaging and therapeutic outcomes [244].

In addition to optimizing shape, size, and surface charge, surface modifications are critical for fine-tuning the pharmacokinetics, biodistribution, and immune interactions of AuNPs. While surface modifications have advanced AuNPs design, their performance in vivo is often compromised by the dynamic and unpredictable nature of the protein corona. This evolving biomolecular layer can override intended surface functionalities, redirect biodistribution, and impair recognition of targeting ligands, regardless of stealth coatings [246, 247]. Additionally, subtle surface features such as charge density or ligand arrangement can unintentionally promote corona formation [248]. Thus, surface engineering must strike a balance between prolonging circulation time and minimizing undesired biological interactions [249].

### Protein corona and immune modulation 

Once in circulation, AuNPs undergo rapid opsonization, meaning they become coated with plasma proteins, leading to the formation of a dynamic protein corona. This corona alters the surface properties of the nanoparticles, significantly increases their hydrodynamic diameter, and enhances phagocytosis by macrophages. This biologically formed corona is distinct from the “native corona,” which forms during synthesis and generally remains stable. In contrast, the “advanced corona” forms in biological environments and is composed of proteins such as immunoglobulins, complement factors, fibrinogen, and other serum components [250]. For instance, Marina et al. observed that the hydrodynamic size of citrate-stabilized AuNPs increased substantially upon plasma incubation, from 33 to 50 nm to 74 and 100 nm, respectively, highlighting the scale of corona-induced transformations [251]. In this altered state, nanoparticles acquire a new biological identity that governs their pharmacokinetics, biodistribution, immune recognition, and cellular uptake.

**Fig. 4:**
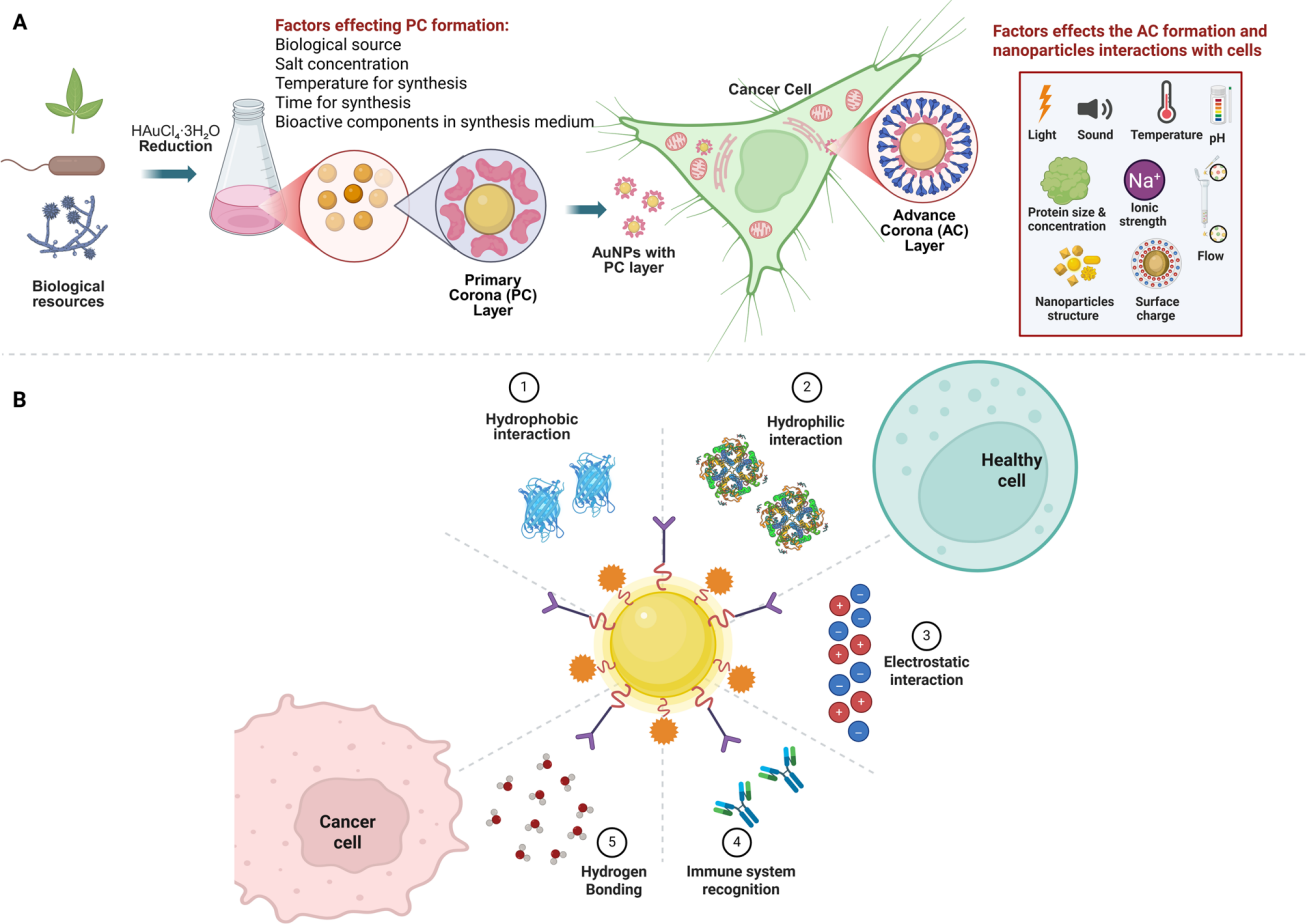
Bio-assisted synthesis and nanoparticle–cell interactions. **A** Biological resources reduce HAuCl₄·3 H₂O to form AuNPs, with bioactive components (e.g., proteins, enzymes, metabolites) forming a primary protein corona (PC). This corona further evolves into an advanced corona (AC) upon interaction with cells. Key factors such as biological source, salt concentration, synthesis conditions, and composition of the medium influence PC formation. Additionally, light, pH, ionic strength, protein concentration, and nanoparticle physicochemical properties modulate AC formation and cellular interactions. **B** Nanoparticles interact with cells through various mechanisms, including hydrophobic, hydrophilic, and electrostatic interactions, immune recognition, and hydrogen bonding, ultimately determining their uptake by cancerous and healthy cells

Interestingly, Glancy et al. reported that nanoparticles smaller than 10 nm may interact with proteins more akin to small molecule protein complexes, where proteins function as carriers and the nanoparticle acts as the payload, making the nanoparticle’s fate highly dependent on the protein’s properties [252]. In addition, Piella et al. showed that the nature of the corona from incomplete to multilayered shells varies strongly with nanoparticle size, further influencing circulation behavior and bioavailability [253]. The advanced corona consists of tightly bound hard layers and loosely associated soft layers, shaped by factors such as pH, ionic strength, temperature, and protein concentration (Fig. 4A) [249]. The corona includes both opsonins (e.g., IgG, C3b, fibrinogen), which promote clearance by the mononuclear phagocyte system (MPS) and dysopsonins (e.g., albumin, clusterin, apolipoproteins), which help evade immune detection and enhance circulation time [254, 255]. Moreover, molecular dynamics simulations indicate that dysopsonins may displace pre-adsorbed opsonins on nanoparticle surfaces, particularly due to higher lateral mobility, leading to a more stable corona and prolonged circulation time [255]. Thus, the balance between these protein classes, shaped by nanoparticle size, shape, and surface chemistry, ultimately influences tumor accumulation and systemic clearance [256].

### Cellular uptake and membrane interactions 

Following tissue extravasation, the cellular pharmacokinetics of AuNPs depend on their interactions with cell membranes and the mechanism of internalization. AuNPs interact with living cells through several mechanisms: hydrophobic interactions between cell membranes and AuNPs surfaces, hydrophilic interactions that stabilize AuNPs dispersion in biological fluids, electrostatic interactions based on charge differences between AuNPs and cell membranes, and hydrogen bonding, which aids in protein binding and cellular uptake (Fig. 4B). These surface-level interactions collectively influence their cellular uptake and fate.

**Fig. 5:**
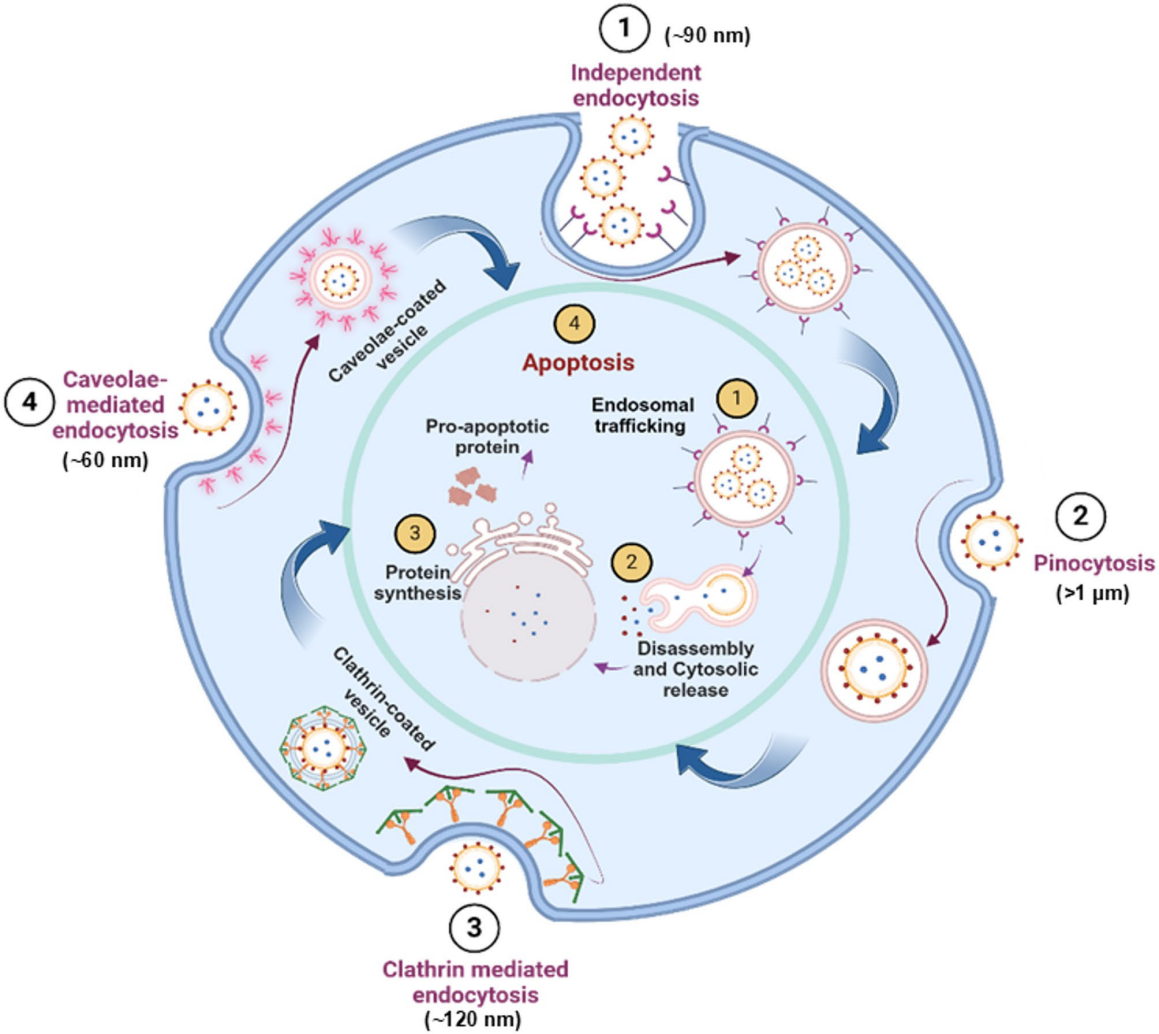
Schematic illustration of cellular uptake and intracellular trafficking pathways of AuNPs. Depending on size and surface characteristics, AuNPs enter cells through various mechanisms, including clathrin- and caveolin-independent endocytosis (~ 90 nm), pinocytosis or macropinocytosis (> 1 μm), clathrin-mediated endocytosis (~ 120 nm), and caveolae-mediated endocytosis (~ 60 nm). Once internalized, they undergo endosomal trafficking and may escape into the cytosol, where they can influence protein synthesis and potentially trigger apoptosis via pro-apoptotic protein expression. These processes collectively govern their therapeutic action and intracellular fate. (Made by using biorender)

In addition, the pharmacology profile also depends on the different ways of endocytosis by which AuNPs enter the cells (Fig. 5) [257]. Pinocytosis, or cell-drinking, represents a non-specific fluid-phase mechanism through which small AuNPs can enter cells. Clathrin-mediated endocytosis, is a receptor-specific process involving clathrin-coated vesicles, often exploited by functionalized or ligand-coated AuNPs. Caveolae-mediated endocytosis involving cholesterol- and sphingolipid-rich membrane domains, enables uptake of certain AuNPs formulations via caveolin-associated receptors. Independent endocytic mechanisms that do not involve classical clathrin or caveolae pathways have also been reported for AuNPs [258].

### Organ-level biodistribution and clearance 

**Fig. 6:**
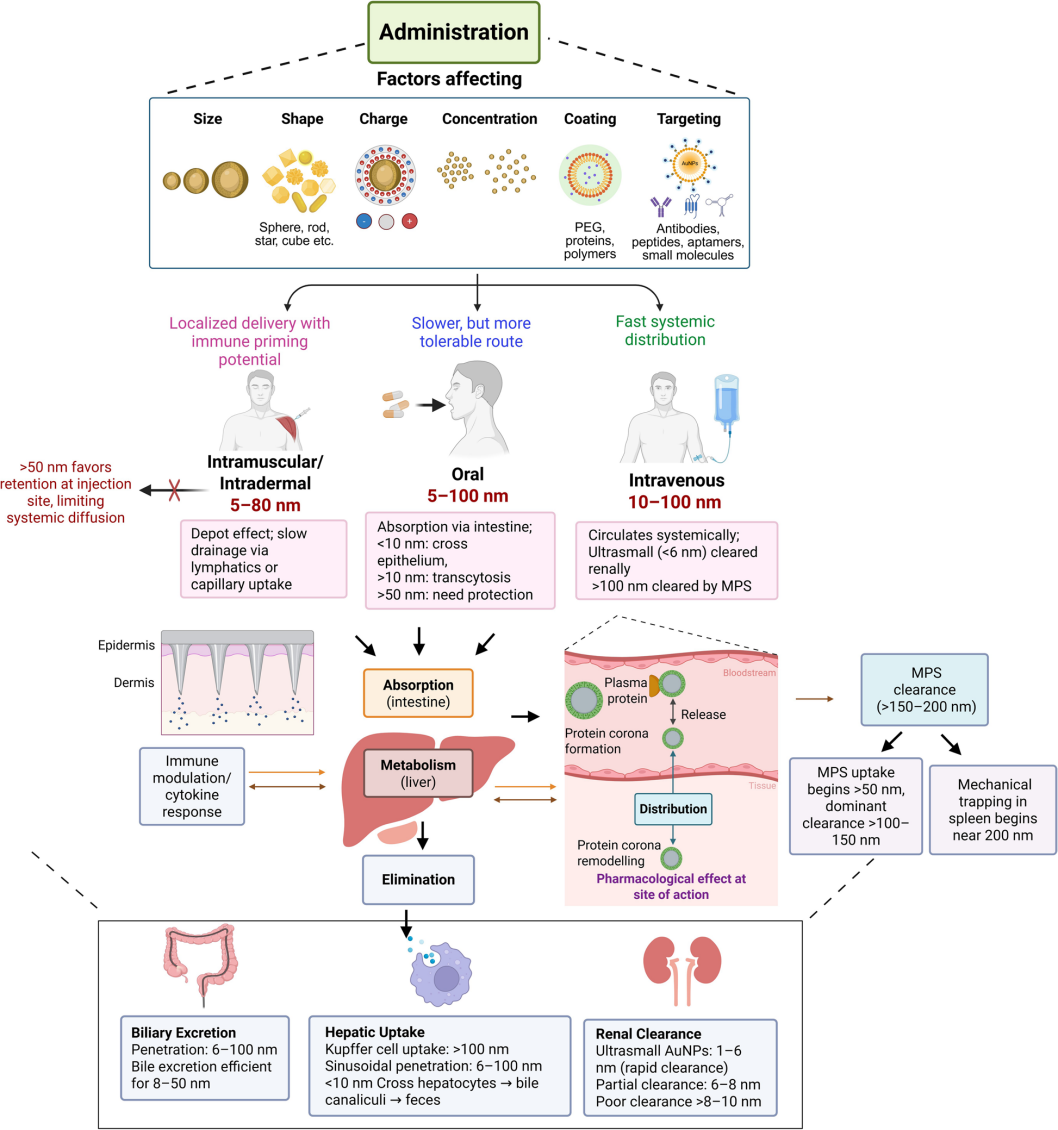
Biodistribution and clearance pathways of AuNPs. AuNPs administered via oral, intramuscular/intradermal (IM/ID), or intravenous (IV) routes exhibit distinct absorption, distribution, metabolism, and elimination profiles influenced by particle size, shape, surface properties, corona, targeting moieties, and delivery route. IM/ID delivery favors localized retention and immune priming. Oral delivery requires transcytosis for >10 nm particles and protective strategies for >50 nm. IV delivery enables rapid systemic circulation, with renal clearance dominant for <6 nm, and MPS uptake above 100 nm. Final elimination occurs via renal, hepatic, or fecal routes based on nanoparticle size and organ-specific uptake mechanisms. (Created by using biorender)

The biodistribution and clearance of AuNPs are governed by a complex interplay of physiological barriers and immune pathways, as summarized in Fig. 6. This includes protein corona formation, tissue-specific accumulation, cellular metabolism, and elimination routes such as hepatic and renal pathways. The lungs play an active role in clearing inhaled AuNPs through two primary mechanisms: mucociliary transport and phagocytosis by alveolar macrophages. Mucociliary clearance helps move particles upward toward the pharynx for elimination, while alveolar macrophages are responsible for removing particles that reach the deeper regions of the lung. The efficiency of these clearance pathways is strongly influenced by nanoparticle size; i.e. larger particles that are more easily recognized and engulfed by macrophages, making them more likely to be retained in the pulmonary environment. Han et al. (2014) investigated the biodistribution of aerosolized AuNPs in Sprague-Dawley rats and found that both small (13 nm) and large (105 nm) particles predominantly accumulated in the lungs post-inhalation. This translocation enables ultrasmall AuNPs to reach downstream organs, including the liver, spleen, brain, and testes, where they may accumulate or undergo further clearance [259].

As AuNPs circulate within the system, the primary clearance mechanism involves the MPS, particularly macrophages located in the liver (Kupffer cells), spleen, and bone marrow. These macrophages recognize opsonized AuNPs and internalize them via phagocytosis, leading to lysosomal accumulation. Liver and spleen uptake is a central feature of MPS activity and represents a dominant route of short-term clearance for most AuNPs. Once sequestered in the liver, AuNPs interact dynamically with hepatic cell populations, including Kupffer cells (resident macrophages), liver sinusoidal endothelial cells (LSECs), hepatocytes, and hepatic B cells, which collectively influence both their biodistribution and long-term fate. Among hepatic cells, Kupffer cells are the dominant site of AuNPs uptake, primarily internalizing particles > 100 nm via phagocytosis facilitated by protein opsonization. In contrast, hepatocytes, being non-phagocytic and located beyond the fenestrated endothelium, contribute minimally to direct uptake. The interaction between AuNPs and hepatic cells is strongly influenced by particle size. Larger engineered nanoparticles (> 200 nm) tend to evade rapid renal clearance but still accumulate in organs such as the liver, spleen, and lungs due to uptake by the MPS. In contrast, medium-sized nanoparticles (20–150 nm) are more efficiently cleared by Hepatic MPS cells, particularly Kupffer cells, which can trap up to 90% of the administered dose [260]. Importantly, once internalized, AuNPs, regardless of size, shape, or surface coating, tend to remain within hepatic cells for extended durations without undergoing significant degradation or exocytosis. Studies using spherical and rod-shaped AuNPs (25–90 nm) demonstrated rapid accumulation in Kupffer cells and LSECs within 24 h post-administration and persistence for up to 47 days [261]. Despite the pronounced intracellular retention, no acute or chronic hepatic toxicity was observed during this period, suggesting a degree of hepatic tolerance, albeit with potential concerns for long-term accumulation [261].

**Fig. 7:**
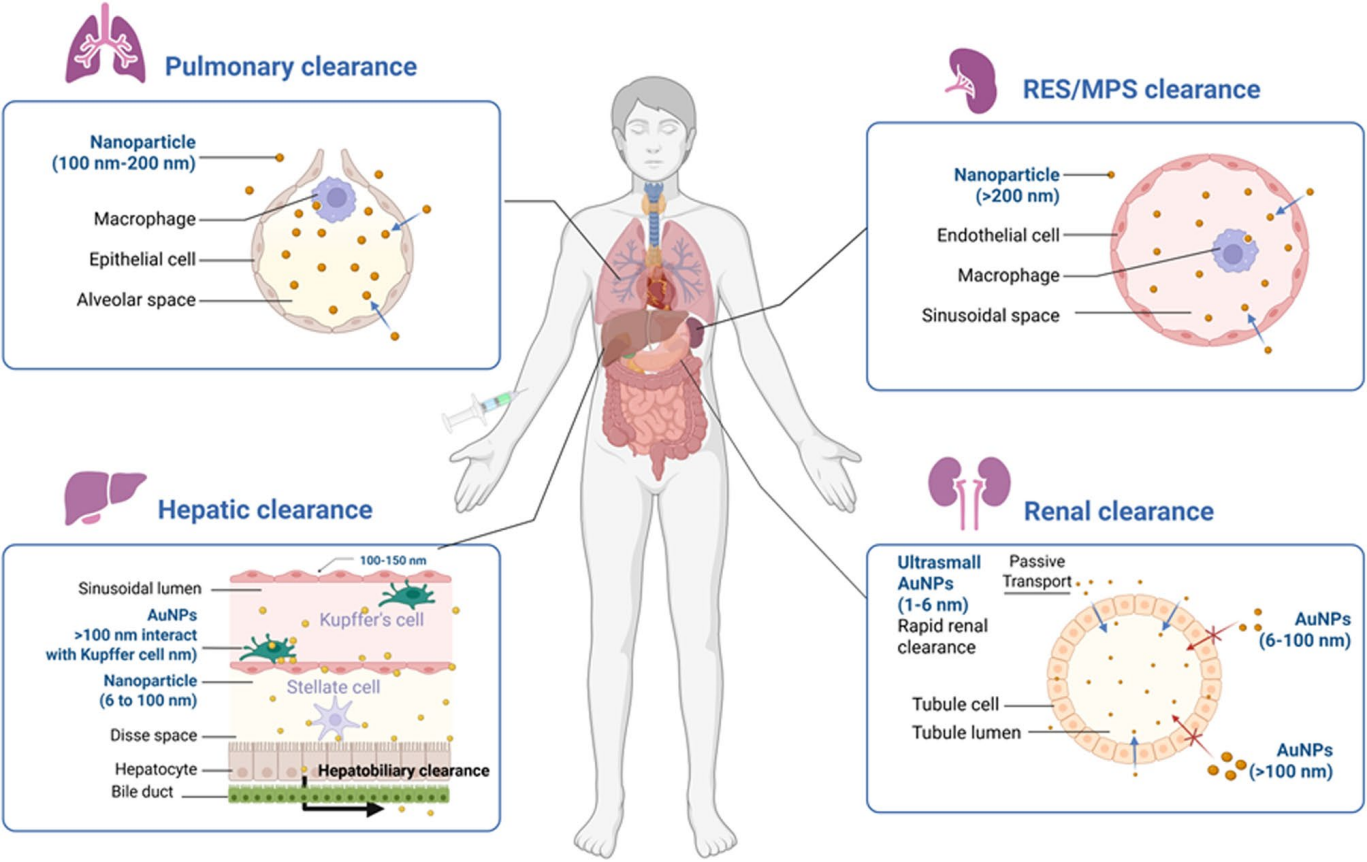
Schematic representation of the biodistribution and clearance of AuNPs based on their size. Pulmonary clearance is highlighted for larger nanoparticles (100–200 nm), which are cleared through the lungs. Renal clearance primarily handles smaller nanoparticles (1–6 nm) via the kidneys. The RES/MPS clearance pathway is shown for medium-sized nanoparticles (> 20 nm), where they are processed by spleen and other immune organs. Hepatic clearance involves Kupffer cells and stellate cells in the Liver for nanoparticles ranging between 20 and 150 nm, as well as smaller particles (< 20 nm)

Within the hepatic microenvironment, markedly reduced blood flow (up to 1,000-fold slower than peripheral circulation), and the specialized sinusoidal architecture promote prolonged nanoparticle residence and sustained interaction with immune cells (Fig. 7). This generates a spatial concentration gradient with higher accumulation occurring near the portal triad, where B and T lymphocytes are localized. B cells, owing to their superior endocytic capacity, are among the earliest immune responders to circulating nanoparticles. Nanoparticles that escape hepatic capture may re-enter systemic circulation via the central vein and recirculate through the liver or other MPS-associated organs before eventual clearance. Similar to Hepatic clearance, the spleen also plays a crucial role in MPS-mediated elimination. In the spleen, elimination is regulated by size, shape, and mechanical flexibility. Particles larger than 200 nm or those with rigid or irregular shapes are typically retained due to poor deformability and their inability to pass through splenic interendothelial slits, which range from approximately 200–500 nm. This leads to mechanical entrapment within the red pulp of the spleen. In contrast, more flexible nanoparticles can better navigate the splenic architecture and are more likely to pass through these narrow gaps. Additionally, the composition of the protein corona, particularly enrichment with immunoglobulins or opsonins, enhances immune recognition and retention within splenic tissues [262].

In contrast, smaller AuNPs (6–100 nm) are more likely to evade Kupffer cell capture and translocate across the sinusoidal endothelium into bile canaliculi, facilitating hepatobiliary elimination and fecal excretion. In parallel, renal clearance serves as a complementary elimination route, particularly for ultrasmall AuNPs (< 8 nm), which can be filtered by the glomerulus due to their small hydrodynamic diameter (i.e., the effective size of nanoparticles in a fluid environment) [263]. These nanoparticles are typically excreted unchanged in the urine, bypassing MPS capture and reducing hepatic load. Renal elimination efficiency is further influenced by surface charge, hydrophilicity, and protein corona composition, which can either promote or hinder glomerular filtration. Notably, PEGylation, means the attachment of polyethylene glycol (PEG) chains to the nanoparticle surface and zwitterionic coatings have been shown to enhance renal clearance by reducing opsonization and minimizing nonspecific interactions in circulation [264]. Xia et al. [265] reported that AuNPs in the 20–50 nm range exhibited the longest circulation time and highest accumulation in the Liver and spleen. In contrast, 5 nm AuNPs were rapidly eliminated via renal filtration but also induced greater cytotoxicity, including apoptosis and necrosis in HepG2 cells, neutrophil activation, and mild hepatotoxicity. Similarly, Piella et al. [253] found that smaller AuNPs were more efficiently absorbed, metabolized, and eliminated, though their enhanced bioavailability may also increase off-target interactions and toxicity.

### Intracellular fate and biotransformation 

Beyond extracellular interactions, intracellular processing, particularly within lysosomes, plays a crucial role in the long-term fate of AuNPs. Once internalized, AuNPs may encounter acidic endosomal compartments, undergo degradation, or escape into the cytoplasm. Additionally, interactions with intracellular organelles such as lysosomes or mitochondria can influence their stability and biological fate [266]. Furthermore, AuNPs may undergo intracellular modifications like redox reactions or ligand exchange, altering their surface chemistry and impacting cellular interactions and responses [267].

Beyond membrane penetration, AuNPs can also access the nucleus due to their nanoscale size, enabling direct interaction with nuclear components. This ability to cross the nuclear envelope allows AuNPs to induce DNA damage and modulate gene expression, particularly in cancer cells, through various mechanisms such as PTT, gene delivery, or radiosensitization. For instance, Özçelik and Pratx developed AuNPs functionalized with RGD and NLS peptides to target cancer cells and their nuclei, significantly enhancing radiation-induced DNA damage. In A549 cells, these AuNPs reduced the required radiation dose for effective treatment and strongly inhibited cell proliferation, demonstrating their potential as nuclear-targeted radiosensitizers [268]. Similarly, Janic et al. demonstrated that 14 nm AuNPs, which preferentially localize to the nucleus, significantly enhanced the efficacy of radiotherapy in a triple-negative breast cancer (TNBC) xenograft model. The nuclear-targeting behavior of these particles led to increased tumor growth delay and improved survival outcomes, along with enhanced immunogenic cell death and macrophage infiltration, suggesting a dual mechanism of action involving both DNA damage and immune activation. Additionally, Tiffany G. et al. (2018) demonstrated successful delivery of DNA-modified AuNPs to targeted brain regions in mice, with 6 nm particles showing Sixfold higher BBB penetration than 14 nm particles. Once inside the brain, such size-optimized AuNPs hold potential for precise intracellular targeting and functional activation [269]. In a non-oncological context, hollow ceria-based nanozymes decorated with AuNPs were employed to deliver miR-486 for treating myocardial ischemia/reperfusion injury (I/RI), resulting in targeted cardiac accumulation, lysosomal escape, ROS scavenging, and reduced cardiomyocyte apoptosis and ventricular remodeling [270].

Some studies have shown that AuNPs are not entirely inert after cellular uptake. For instance, Balfourier et al. demonstrated a two-step biotransformation process in which AuNPs undergo size-dependent degradation mediated by ROS from NADPH oxidase, followed by recrystallization into biomineralized nanostructures resembling historical aurosomes [271, 272]. This process, likely regulated by metallothioneins, reveals a form of intracellular gold metabolism, with implications for bioactivity, clearance, and potential toxicity, especially under conditions of repeated or long-term exposure [271].

### Long-term retention, immune effects, and biodegradable strategies

Several in vivo studies report conflicting evidence regarding AuNPs clearance, particularly due to their non-biodegradable nature, which allows them to persist in tissues for extended durations. This prolonged retention, especially in MPS-associated organs like the liver and spleen-raises concerns about long-term immunological effects and systemic toxicity [273]. Parameters such as size, shape, surface charge, and ligand conjugation critically influence biodistribution and clearance pathways. Larger (> 10 nm), non-degradable AuNPs tend to accumulate in organs for weeks to months, potentially disrupting normal immune function.

One consequence of chronic exposure is MPS saturation, where macrophages become overloaded, impairing their ability to clear subsequent nanoparticles or pathogens. MPS saturation, often observed even after a single high-dose nanoparticle administration, can temporarily impair macrophage-mediated clearance of subsequent particles or pathogens, though this effect is not always sustained [274]. This transient saturation can significantly alter nanoparticles pharmacokinetics, prolong systemic circulation and modulate immune surveillance. A recent study identified a dose threshold (~ 1 trillion nanoparticles in mice) above which Kupffer cells become saturated, leading to nonlinear reductions in Hepatic clearance and enhanced tumor accumulation of up to 12% of the injected dose [274]. Moreover, AuNPs exposure has been linked to macrophage polarization, not only toward classical M1/M2 phenotypes but also toward dysfunctional or hybrid states with impaired responsiveness to tumor antigens or pathogen-associated molecular patterns (PAMPs) [275]. Persistent interactions with immune cells may desensitize toll-like receptor (TLR) pathways, reduce antigen presentation by dendritic cells, and lead to splenic lymphoid atrophy, collectively compromising adaptive immunity.

These immune modulations often remain undetected in short-term toxicological assessments that focus primarily on acute cytotoxicity or organ burden. However, for nanomedicines requiring repeated administration such as in cancer or chronic inflammatory diseases, these subtle but chronic immune alterations could undermine therapeutic efficacy, provoke adverse events, or contribute to systemic immune exhaustion. Nonetheless, several studies have shown that AuNPs, even at high doses, exhibit minimal systemic toxicity despite pronounced accumulation in liver sinusoidal cells [274]. In another study, Qi et al. (2024) demonstrated that even renal-clearable AuNPs are susceptible to hepatic modification. Using IRDye800CW–Au₂₅ nanoclusters, which avoided liver uptake and protein binding, they found that hepatic glutathione gradually cleaved surface ligands during circulation [261]. This process enabled a urinary biotransformation index (UBI) correlating with liver redox status offering a potential noninvasive diagnostic tool for hepatic function [276]. Furthermore, repeated AuNPs accumulation has been associated with low-grade, non-resolving inflammation, leading to fibrotic remodeling, extracellular matrix deposition, and disrupted sinusoidal structure, all of which can impair hepatic function and drug metabolism. These findings underscore the limitations of relying solely on short-term toxicity endpoints. Comprehensive long-term safety assessments are essential to evaluate not only biodistribution and clearance, but also immune remodeling, histopathological changes, and the recovery of immune competence after nanoparticle exposure.

To address these concerns, PEGylation, has emerged as a gold standard for enhancing systemic stability [277]. PEG forms a hydrophilic barrier that minimizes nanoparticle aggregation, reduces protein opsonisation, and limits macrophage uptake, thereby diminishing immune recognition and extending circulation time. These effects collectively improve bioavailability and tumor accumulation relative to uncoated AuNPs [278, 279]. For example, Khlebtsov et al. [280] showed that PEGylated AuNPs remained in Hepatic and splenic tissues for up to 28 days post-injection. Although early histopathological changes such as hepatocyte vacuolation and splenic apoptosis diminished over time, delayed shifts in liver injury markers and lipid metabolism were observed, indicating subclinical toxicity. The performance of PEGylated AuNPs depends strongly on both PEG chain length and surface density. Nanoparticles with PEG densities below 0.2 molecules per nm² are insufficient to prevent opsonisation and rapid MPS-mediated clearance [249], while coatings exceeding ~ 20 PEG chains (5 kDa each) per 100 nm² consistently prolong circulation, independent of nanoparticle size. Quach et al. also showed that PEGylation bolsters the stability of AuNPs by minimizing opsonin interactions and facilitating prolonged systemic circulation [277] Wang et al. reported that PEG chains of ~ 13.8 nm in length (corresponding to 5000 Da) optimally reduced protein adsorption and macrophage uptake, resulting in prolonged circulation, enhanced tumor accumulation, and improved therapeutic efficacy [281]. These findings position PEG5000 as a particularly effective coating for systemic applications.

Beyond PEG, other surface chemistries have demonstrated similar benefits. Zwitterionic polymers and low-fouling biomolecules such as glutathione, lysine, and cysteine also reduce nonspecific serum protein interactions and extend nanoparticle circulation [282]. Additionally, synthetic polymers like N-(2-hydroxypropyl)methacrylamide (HPMA) copolymers can mask nanoparticle surfaces and improve in vivo performance by further limiting opsonin binding [283]. Moreover, surface functionalization with targeting ligands, including antibodies, peptides, and small molecules can improve tumor specificity by directing AuNPs to receptors involved in cancer cell proliferation, metabolism, or angiogenesis. This strategy also holds promises for immunomodulation. For example, functionalized AuNPs bearing immunoregulatory peptides have been used to selectively target regulatory T cells, demonstrating therapeutic benefits in autoimmune disease models [284]. Collectively, these surface engineering strategies not only improve circulation time and biodistribution but also enable precision targeting and reduce off-target effects, reinforcing the value of surface chemistry in the rational design of clinically translatable AuNPs-based nanomedicines.

Recent efforts have also focused on engineering biodegradable AuNPs using environmentally responsive linkers [285]. An in vivo study demonstrated that, despite prolonged hepatic accumulation, well-characterized and endotoxin-free AuNPs did not induce liver toxicity in mice up to seven weeks post-administration [261]. These particles were rapidly internalized by liver sinusoidal endothelial cells and Kupffer cells; however, liver enzyme levels remained within normal ranges, supporting their long-term biocompatibility even within primary clearance organs [261]. Notably, glutathione-sensitive or disulfide-bonded assemblies have shown promise for enabling controlled degradation of AuNPs structures in response to intracellular redox conditions. This is particularly effective in tumor cells or phagocytic immune cells, where glutathione levels are elevated [285]. Such redox-responsive designs allow AuNPs to disassemble into smaller, renal-clearable fragments, thereby facilitating elimination through the kidneys and reducing persistent accumulation in MPS-associated tissues [286]. For example, a recent study demonstrated that 3-nm glutathione-coated AuNPs, capable of renal clearance, achieved superior tumor targeting and reduced organ accumulation in a murine glioblastoma model compared to larger, non-clearable counterparts [287]. In line with these advancements, Loynachan et al. (2019) developed ultrasmall gold nanocluster-based, protease-responsive nanosensors that leverage the peroxidase-mimicking catalytic activity of AuNPs for rapid, noninvasive disease detection [288]. In a colorectal cancer mouse model, these nanosensors produced a 13-fold increase in urinary colorimetric signal within just one hour. Importantly, they exhibited complete renal and hepatic clearance within four weeks and showed no observable toxicity [288]. These multifactorial dependencies between nanoparticle properties and biological systems must be thoroughly understood and integrated into design pipelines, especially as many AuNPs formulations move from preclinical validation into clinical testing.

## Real-world applicability: AuNPs versus other nanocarriers

Based on recent survey, (www.researchandmarkets.com, last accessed on 11/06/2025), the market for AuNPs is expected to rise to USD 1.11 billion by 2029 from the current value of USD 0.50 billion. This report also suggests that this expected rise in the AuNPs market is from the pharmaceutical and healthcare industries, as well as from the personal care and cosmetic industries, followed by electronics and electrical industries. This proves that, in future, AuNPs will be one of the prime candidates for the diagnosis and therapy of several diseases. To understand the real-world applicability of AuNPs, they can be compared with other inorganic nanoparticles (e.g., iron oxide nanoparticles) as well as with organic nanoparticles (e.g., liposomes, polymeric nanoparticles, lipid nanoparticles, and proteinaceous nanoparticles). In the case of other inorganic nanoparticles, iron oxide nanoparticles, silver nanoparticles, and silica nanoparticles have gained quite attention. Many of these nanoformulations are already in clinical trials. Iron oxide, being the most successful among them, has entered the market for imaging as well as treatment purposes. For example, Dexferrum^®^ and Feraheme^®^ have been approved by the FDA for the treatment of iron deficiency in CKD and iron-deficient anemia, respectively [289, 290]. As compared to these marketed iron oxide nanoparticles, AuNPs are still a step behind and have entered clinical trials. Nonetheless, AuNPs show comparable properties concerning bioimaging and treatment. In recent years, hybrid nanoassemblies of AuNPs and iron oxide nanoparticles have been explored. Such hybrid nanoassembly was shown to have excellent pharmacokinetic properties along with theranostic applications [291, 292]. On the other hand, silica nanoparticles loaded with either fluorophore or radiolabeled peptide are in clinical trials for lymph node mapping as well as brain tumor imaging [289]. Whereas the hybrid gold-silica nanoshells; also terms as Auroshell^®^, have received approval for performing clinical trials for prostate cancer therapy [293], https://nanospectra.com/, last accessed on 16/07/2025). These reports prove the excellent and versatile applicability of AuNPs with or without other inorganic nanoparticles. At present, no report explicitly shows a comparative study of optical, thermal, and electronic properties of gold, iron oxide, and silica nanoparticles. But size and shape-dependent modulation of AuNPs allows the user to yield the nanoformulations with desirable contrast power, encapsulation of one or more bioactive agents, and desirable pharmacokinetic properties. Furthermore, the capacity of gold to get merged with other inorganic nanoparticles offers better options to obtain dual-action hybrid nanostructures for biomedical use.

Organic nanotherapeutics in the form of liposomes (Doxil^®^), protein nanoparticles (Abraxane^®^), and polymeric micellar drug delivery systems (Genexol^®^ PM) have been approved by US-FDA for quite a few years [294]. As compared to these nanotherapeutics, very few AuNPs have received US-FDA approval. The majority of the gold nanoformulations are still under clinical trial. Polymeric, lipidic, and proteinaceous drug delivery systems lack specific optical, thermal, and electrical properties, where AuNPs stand out as the best option while being inert and biocompatible on equal grounds with polymeric, lipidic, and proteinaceous systems. Hence, in specific diseases such as cancer or cardiovascular diseases, AuNPs show great potential. Similar to inorganic nanoparticles, the AuNPs can also be merged with other organic nanoparticles to improve the applicability of hybrid nano-systems in biomedical field. For example, lipid-AuNPs nanoclusters functionalized with antibodies have shown promises in ultrasensitive and selective diagnosis of gangliosidosis. In this report, lipid components offered better stability and penetration in the cells, whereas AuNPs aided SERS-mediated detection [295]. In another report, Li et al. have shown utility of protein-AuNPs nanoclusters in MRI and NIR assisted imaging and theranostic applications. Authors used keratin-AuNPs nanoclusters with 6.5-fold higher fluorescence and excellent stability (~ 4 months) and biocompatibility in animal models. Using doxorubicin as a model anticancer drug, authors showed successful drug delivery using these nanoclusters [296]. Among organic nanoparticles, polymers have been extensively used along with AuNPs to make hybrid nanocomposites for various biomedical applications [297, 298]. Polymers offer various attributes to AuNPs in hybrid systems, such as stability, biological activity, longer circulation time, slow degradation, enhanced cell penetration, and biocompatibility. So, to conclude, AuNPs play crucial roles in hybrid inorganic or organic nanomaterials that are employed in biomedical applications. No other nano-system offers versatility in modulating size and shape as easily as AuNPs. However, we believe that owing to limitation of functional groups available on the surface of AuNPs, the surface functionalization with the help of bioconjugation reactions becomes a difficult task. In this regard, the polymeric/lipidic/proteinaceous nano-systems offer great advantage because of the availability of variety of functional groups in their native as well as chemically modified structure. Nonetheless, several reports have shown successful surface functionalization of AuNPs using antibodies, peptides, oligonucleotides, and polymers in recent times [299–301]. Image-guided therapy has received quite an attention in recent years due to its advantages such as real-time monitoring of diseased tissue, spatio-temporal control on treatment modality, high specificity, and high sensitivity. AuNPs offer special optical characteristics owing to their variable geometrical features that make them a perfect candidate for use in image-guided therapy. In the case of other nano-systems, to be eligible for real-time imaging, a special contrast agent must be loaded onto the nano-systems. Hence, AuNPs offer excellent advantages as compared to other nano systems. Furthermore, the capability to conduct heat and electricity makes them unique ideal candidates in case of thermal therapy of cancer as well as in the treatment of CVDs, respectively. Polymeric/lipidic/proteinaceous nano-systems do not offer such applicability. In few other aspects, such as co-delivery of two bioactive agents or drugs and spatio-temporal control on their release, the AuNPs show comparable results with other nano-systems. Despite significant advancements in the field of AuNPs, they remain relatively underexplored in clinical settings. Nevertheless, several gold-based nanoformulations are currently undergoing clinical trials, indicating the strong likelihood of their eventual entry into the healthcare market. The following section discusses their clinical applications, translational outcomes, and the remaining challenges in fully realizing their therapeutic potential.

## Clinical trials and regulatory considerations

While some AuNPs-based therapeutics have encountered setbacks due to complex pharmacokinetics, such as rapid clearance by the MPS, renal elimination, or reduced targeting from ligand masking, it is important to highlight that not all clinical trials have failed to translate. In fact, several AuNPs-based interventions have demonstrated strong safety and efficacy profiles and have either progressed to late-stage clinical trials or received regulatory approval for clinical use (Table 3). The first clinical trial involving AuNPs began in 2005, led by CytImmune Sciences, testing Aurimune (CYT-6091), which used AuNPs conjugated with TNF-α to target tumors via the EPR effect, reducing systemic toxicity. The Phase I trial (NCT00356980, 2006–2009) demonstrated that TNF-bound AuNPs were well-tolerated in patients with advanced solid tumors, allowing for higher doses of TNF-α with fewer side effects. A parallel cohort study (NCT00436410) confirmed effective nanoparticle localization in tumor tissues across various cancers, including colorectal, pancreatic, and breast cancers. Results from this clinical trial showed enhanced drug delivery to tumors with reduced systemic toxicity, demonstrating promising antitumor effects [302]. Another clinical study, NCT03020017 Phase 0 trial evaluated the safety and efficacy of NU-0129, a spherical nucleic acid therapeutic, in patients with recurrent glioblastoma multiforme or gliosarcoma. NU-0129 consisted of nucleic acids arranged on the surface of small spherical AuNPs, which enabled it to cross the BBB and target the Bcl2L12 gene, a gene that promoted tumor growth by preventing apoptosis. The trial, completed in 2020, showed that NU-0129 was well-tolerated, with only mild, reversible side effects like hypophosphatemia and lymphopenia. Importantly, the AuNPs-based spherical nucleic acid successfully accumulated in tumor tissues and reduced Bcl2L12 expression. However, further trials are needed to confirm its long-term therapeutic efficacy [303]. Additionally, Nanospectra Biosciences, Inc. conducted clinical trial using AuroShell nanoparticles for various cancers between 2014 and 2020, with mixed outcomes [304]. AuroShell was a nanoparticle consisting of a silica core coated with a thin layer of gold, designed for PTT. When exposed to NIR light, these nanoparticles generate heat, allowing for target tumor ablation. The prostate cancer trial (NCT02680535), completed in 2020, showed promising results with successful tumor ablation and minimal side effects. The NCT04240639 trial, which began in 2020, was still recruiting. The lung cancer trial (NCT01679470) was terminated in 2014 for strategic reasons, and the head and neck cancer trial (NCT00848042), completed in 2014, demonstrated safety and feasibility, though detailed efficacy results were not published. Another clinical trial, NCT04907422 (2021), explored a new diagnostic and prognostic method using CD24-Gold Nanocomposites for detecting cancer stem cells in salivary gland tumors. This approach aimed to improve the sensitivity and specificity of detecting tumors such as carcinoma ex pleomorphic adenoma and pleomorphic adenoma through real-time quantitative PCR. The study successfully demonstrated that CD24-Gold Nanocomposites offered enhanced accuracy compared to non-conjugated CD24, marking a significant step forward in the early detection of cancer stem cells [305].

**Table 3:** Clinical trials involving AuNPs across various medical fields, detailing trial ids, study titles, targeted conditions, phases, and key outcomes, highlighting their potential in diagnostics, drug delivery, and therapeutic applications

Trial ID	Study Title	Condition Studied	Trial Phase	Outcome
NCT00356980	Aurimune (CYT-6091) Phase I	Advanced Solid Tumors	Phase I	Well-tolerated, reduced TNF-α toxicity
NCT00436410	Aurimune Tumor Localization Study	Tumor Localization (Colorectal, Pancreatic, Breast)	Early Phase 1, Completed	Effective localization of nanoparticles in tumors
NCT03020017	NU-0129 for Glioblastoma	Recurrent Glioblastoma Multiforme or Gliosarcoma	Phase 0	Safe, reduced Bcl2L12 expression in tumors
NCT02680535	AuroShell (AuroLase) Nanoparticles for Prostate Cancer	Prostate Cancer	Completed	Successful tumor ablation, minimal side effects
NCT04240639	AuroShell Nanoparticles for Cancer	Various Cancers	Recruiting	Still recruiting; no interim efficacy data reported yet
NCT01679470	AuroShell for Lung Cancer	Lung Cancer	Terminated	Terminated for strategic reasons
NCT00848042	AuroShell for Head & Neck Cancer	Head & Neck Cancer	Completed	Safe, feasible, efficacy data not published
NCT04907422	CD24-Gold Nanocomposites in Salivary Gland Tumors	Salivary Gland Tumors	Completed	Enhanced cancer stem cell detection
NCT03815916	CNM-Au8 for Parkinson’s Disease (REPAIR-PD)	Parkinson’s Disease	Phase 2	Improved brain energy metabolism
NCT03993171	CNM-Au8 for Multiple Sclerosis (REPAIR-MS)	Multiple Sclerosis	Phase 2	Safe; interim data shows improved energy metabolism biomarkers
NCT03536559	CNM-Au8 for MS (VISIONARY-MS)	Multiple Sclerosis	Ongoing	Ongoing, neuroprotection potential
NCT04081714	CNM-Au8 for ALS (REPAIR-ALS)	Amyotrophic Lateral Sclerosis	Expanded Access	Access-based support for MS patients; CNM-Au8 targets cellular bioenergetics
NCT01270139	Plasmonic Nanophotothermal Therapy of Atherosclerosis (NANOM-FIM)	Stable Angina, Multivessel CAD, Atherosclerosis, Heart Failure	Randomized, Controlled (Completed)	Feasible AuNP-based therapy; reduced restenosis in low SYNTAX-score CAD; 180 patients
NCT02219074	Sebacia Microparticles for Acne	Acne Vulgaris	Completed	60% acne lesion reduction in 6 months
NCT02837094	C19-A3 AuNPs for Type 1 Diabetes	Type 1 Diabetes	Phase I	Safe, potential for prolonged immune modulation
NCT05113862	naNO-COVID AuNP Vaccine	COVID-19	Phase I	Safe; shows memory T-cell response as booster candidate
NCT04935801	naNO-DENGUE AuNP Vaccine	Dengue	Phase I	Safe, prevented ADE in dengue
NCT05816512	Pelargonium-AuNPs for Oral Hygiene	Gingivitis	Ongoing	Reduced bacterial pathogens, improved oral hygiene
NCT05884515	AuNPs-Based Rapid COVID-19 Test	COVID-19 Diagnosis	Ongoing	Fast and affordable antigen test
NCT05633446	T-cell Priming COVID-19 Vaccine with AuNPs	COVID-19 Vaccine	Ongoing	Long-lasting cellular immunity against COVID-19

AuNPs were also investigated in clinical trials as potential treatments for neurodegenerative diseases. One notable example was CNM-Au8, a therapeutic based on nanocrystalline gold, consisting of 13-nm gold nanocrystals in a drinkable bicarbonate solution. It was designed to target neurodegenerative conditions such as ALS, PD, and MS. CNM-Au8 enhanced the bioenergetic capacity of cells by improving mitochondrial function and increasing the NAD+/NADH ratio, which supported ATP production. In the REPAIR-PD trial (NCT03815916) for PD, completed in 2020, CNM-Au8 demonstrated improvements in brain energy metabolism without significant adverse effects. Similarly, the REPAIR-MS trial (NCT03993171), a Phase 2 study, was initiated to evaluate the effects of CNM-Au8 on patients with MS, focusing on improving brain energy metabolism and reducing oxidative stress-both critical factors in neurodegenerative diseases. CNM-Au8 worked by enhancing mitochondrial function and supporting neuronal survival under stress. The trial is still recruiting participants, and its outcomes are expected to provide valuable insights into neuroprotection and potential treatments for MS. Ongoing trials, such as VISIONARY-MS (NCT03536559), continue to explore CNM-Au8’s broader therapeutic potential for MS, with the hope of advancing effective treatments for neurodegenerative diseases [201].

In addition to its use in neurodegenerative diseases, AuNPs have been explored in clinical trials for cardiovascular conditions. The NCT01270139 trial involves gold nanoshells with a 60–70 nm Silica core and a 15–40 nm gold shell, used for the photothermal ablation of atherosclerotic plaques [151]. This technique employs NIR light to heat the gold nanoshells, causing localized heating that destroys plaques. Early results showed that this PTT effectively reduced plaque size and improved blood flow with no significant adverse effects, showing promise as a minimally invasive treatment for atherosclerosis. Moreover, AuNPs trials were also conducted in dermatology. The Sebacia microparticles trial (NCT02219074), initiated in 2015, employed gold and Silica nanoparticles activated by a 1064 nm Nd laser to treat acne vulgaris. This PTT has received CE Mark approval in Europe and FDA clearance in the United States, establishing one of the first commercialized AuNPs-based dermatological treatments. These nanoparticles, activated by laser to target sebaceous glands, offer a non-antibiotic, minimally invasive solution for acne vulgaris and have been adopted in dermatology clinics [306]. AuNPs have also entered the world of diabetes. The Phase 1 trial NCT02837094 (EE-ASI-1) investigated the use of C19-A3 AuNPs-a proinsulin peptide conjugated to ultrasmall AuNPs-as an immunotherapeutic strategy to modulate the autoimmune response in Type 1 Diabetes. This innovative approach involved intradermal delivery via microneedles, targeting the prevention of insulin-producing cell destruction. The findings of this study demonstrated that the treatment was well-tolerated, with only mild local skin reactions and no major safety concerns reported. While primarily designed to assess safety, the persistence of the nanoparticles in the skin suggests the potential for prolonged immune modulation [307]. In addition to their use in diabetes and neurodegenerative diseases, AuNPs are being explored in vaccine development. The naNO-COVID trial (NCT05113862) demonstrated the safety of an AuNP-based vaccine that primes T-cell responses against SARS-CoV-2, offering a scalable approach to combat viral infections [308]. Similarly, the naNO-DENGUE trial (NCT04935801) showed safety and tolerability for a T-cell-targeted dengue vaccine designed to avoid antibody-dependent enhancement, a common complication in dengue treatments [309]. Both trials highlight potential of AuNPs for generating cell-mediated immunity, presenting a new path for effective viral vaccines.

NCT05816512 is exploring the antimicrobial efficacy of AuNPs derived from *Pelargonium Graveolens* leaves in a mouthwash for children with gingivitis, aiming to reduce bacterial pathogens like *Streptococcus mutans* and *Candida albicans* through improved oral hygiene [310]. NCT05884515, sponsored by IDX20 Inc., focuses on using AuNPs-based rapid antigen tests to detect SARS-CoV-2, aiming to provide an affordable, fast diagnostic tool for COVID-19 screening. NCT05633446, led by Emergex Vaccines Holding Ltd., evaluates a T-cell priming COVID-19 vaccine using AuNPs, which aims to elicit long-lasting cellular immunity against the virus and address variant challenges. Meanwhile, NCT03993171 (REPAIR-MS) investigates the effects of CNM-Au8, a gold nanocrystal therapeutic, on improving brain energy metabolism and reducing oxidative stress in MS patients [201]. Thus, AuNPs have demonstrated remarkable versatility in various clinical applications, spanning diagnostics, immune enhancement, and cellular bioenergetics. They can be administered through multiple routes-intravenous, intramuscular, oral, or local tailored to diseases like type I diabetes, atherosclerosis, acne vulgaris, ALS, MS, and PD. This adaptability underscores the potential of AuNPs to be customized based on clinical needs, enhancing their therapeutic efficacy across a broad range of medical conditions. These success stories illustrate that AuNPs-based therapeutics can overcome pharmacokinetic and biological barriers through smart engineering, such as PEGylation for extended circulation, optimized particle size for EPR exploitation, and precise ligand conjugation for target specificity.

As AuNPs advance toward potential clinical applications, navigating the regulatory landscape is critical to ensure their ethical use, safety, and transformative potential in patient care. Regulatory bodies, such as the US-FDA and the European Medicines Agency (EMA), play pivotal roles in scrutinizing the safety profile of any drugs, nanomaterials, including AuNPs [311]. The evaluation extends beyond traditional assessments, given the unique physicochemical properties of AuNPs that can vary based on size, shape, and surface modifications. These evaluations focus on understanding the interactions between AuNPs and biological systems, ensuring the safety of their use. Long-term safety assessments are emphasized, not just short-term effects, as the accumulation of nanoparticles in the body poses potential risks that must be thoroughly examined. Preclinical data, particularly in cell and animal models, are critical for identifying the optimal dosage, dosing frequency, and therapeutic window to avoid adverse effects. Regulators prioritize these studies to establish a comprehensive safety profile for AuNPs-based therapies. In addition to safety, regulatory authorities require robust evidence of clinical efficacy. Well-designed clinical trials, with controls, randomization, and blinding, are essential to demonstrate the therapeutic or diagnostic benefits of AuNPs. Clinical endpoints must be carefully defined to measure the intended outcomes, whether for treatment, imaging, or drug delivery. Moreover, adherence to Good Manufacturing Practices (GMP) standards is a foundation for regulatory considerations for AuNPs-based interventions. GMP standards ensure the consistent production of AuNPs, minimizing batch-to-batch variations and ensuring that the nanoparticles meet quality requirements for clinical use [312]. This framework guarantees the purity, safety, and reliability of AuNPs-based products, facilitating their translation from laboratory research to clinical practice. Regulatory approval also demands a deep understanding of how AuNPs behave in the human body, including their biodistribution, clearance, and potential toxicity. A thorough risk-to-benefit analysis is mandatory for assessing the overall safety and efficacy of AuNP-based interventions.

Although no AuNPs-based therapeutics have yet received full regulatory approval from agencies such as the US FDA or EMA, a growing number of clinical trials have demonstrated encouraging safety profiles, therapeutic potential, and disease-specific versatility. Regulatory progress has been slowed by the need for more rigorous data on long-term safety, biodistribution, clearance mechanisms, and consistent efficacy across patient populations. While pharmacokinetic factors continue to challenge many AuNPs candidates, a growing body of clinical evidence and market entries suggest that these barriers can be successfully addressed. With continued alignment between scientific advances and regulatory expectations, AuNPs are well-positioned to emerge as a transformative platform in oncology, neurology, dermatology, infectious diseases, and beyond.

## Conclusion and future perspective

While AuNPs have been widely celebrated for their multifunctionality in biomedical applications ranging from drug delivery to diagnostics, their clinical translation remains limited. Despite decades of promising preclinical data, few AuNPs-based formulations have advanced beyond early-stage trials, and none have yet received full regulatory approval for routine clinical use. This gap between bench and bedside underscores the complexity of biological systems and the challenges of navigating them with engineered nanomaterials. A critical issue is the disconnect between preclinical models and human physiology, which often results in misleading efficacy and safety profiles. Most AuNPs designs are optimized for animal models that poorly mimic the immune responses, tumor heterogeneity, or clearance mechanisms observed in humans. Overreliance on the EPR effect and underestimation of immune clearance have contributed to inconsistent clinical outcomes. To move forward, the field must shift from purely engineering-driven approaches to system-aware design, where AuNPs are developed in close alignment with disease biology, immune landscape, and patient-specific variability. This includes rethinking nanoparticle size, charge, and surface modifications not just for targeting efficiency, but also for predictable pharmacokinetics and minimal off-target effects. Furthermore, the integration of artificial intelligence (AI), machine learning (ML), and high-resolution omics technologies offers a promising path to rational nanoparticle design. These tools can accelerate the discovery of novel targeting ligands, predict nanoparticle-host interactions, and refine patient stratification for clinical trials. 

## Data Availability

No datasets were generated or analysed during the current study.
